# The path-label reconciliation (PLR) dissimilarity measure for gene trees

**DOI:** 10.1186/s13015-025-00284-8

**Published:** 2025-08-19

**Authors:** Alitzel López Sánchez, José Antonio Ramírez-Rafael, Alejandro Flores-Lamas, Maribel Hernández-Rosales, Manuel Lafond

**Affiliations:** 1 Department of Computer Science, University of Sherbrooke, 2500 Bd de l’Université, J1K2R1, Sherbrooke, QC Canada; 2 Department of Genetic Engineering, Center for Research and Advanced Studies of the National Polytechnic Institute, Libramiento Norte Carretera Irapuato León Kilómetro 9.6, Carr Panamericana Irapuato León, 36821 Irapuato, Guanajuato Mexico; 3 Department of Computer Science and Interdisciplinary Center for Bioinformatics, University of Leipzig, Härtelstr. 16-18, 04107 Leipzig, Saxony Germany; 4 Max Planck Institute for Mathematics in the Sciences, MPI MIS, Inselstraße 22, 04103 Leipzig, Saxony Germany

**Keywords:** Reconciliation, Gene trees, Species trees, Evolutionary scenarios

## Abstract

**Background:**

In this study, we investigate the problem of comparing gene trees reconciled with the same species tree using a novel semi-metric, called the Path-Label Reconciliation (PLR) dissimilarity measure. This approach not only quantifies differences in the topology of reconciled gene trees, but also considers discrepancies in predicted ancestral gene-species maps and speciation/duplication events, offering a refinement of existing metrics such as Robinson-Foulds (RF) and their labeled extensions LRF and ELRF. A tunable parameter $\alpha$ also allows users to adjust the balance between its species map and event labeling components.

**Our contributions:**

We show that PLR can be computed in linear time and that it is a semi-metric. We also discuss the diameters of reconciled gene tree measures, which are important in practice for normalization, and provide initial bounds on PLR, LRF, and ELRF. To validate PLR, we simulate reconciliations and perform comparisons with LRF and ELRF. The results show that PLR provides a more evenly distributed range of distances, making it less susceptible to overestimating differences in the presence of small topological changes, while at the same time being computationally efficient. We also apply our measure to evaluate the set of possible rootings of gene trees against a gold standard, and demonstrate that our measure is better at distinguishing one best gene tree among multiple candidates. Furthermore, our findings suggest that the theoretical diameter is rarely reached in practice. The PLR measure advances phylogenetic reconciliation by combining theoretical rigor with practical applicability. Future research will refine its mathematical properties, explore its performance on different types of trees, and integrate it with existing bioinformatics tools for large-scale evolutionary analyses. The implementation of the PLR distance is available in the open-source PyPI package parle: https://pypi.org/project/parle/.

## Introduction

During evolution, it is well-known that genes can be duplicated, lost, and transferred, resulting in evolutionary scenarios that differ from the history of the species that contain them. Gene trees can therefore be discordant with their species trees, and *reconciliation* aims to infer the macro-evolutionary events that explain the discrepancies. Several models have been proposed to achieve this task, allowing duplications and losses [1–9], horizontal gene transfer [10–19], incomplete lineage sorting [20–25], and others (see e.g. [26–32]). In addition, some of these models support segmental events that affect multiple genes at once [33–37], and some approaches infer histories based on parsimony whereas others are probabilistic [38–40].

This variety of reconciliation models and algorithms is accompanied by a large diversity of software and tools to reconcile gene trees with species trees (examples include NOTUNG [5], DLCoal [22], RANGER-DTL [41], ecceTERA [42], Jane [43]). Most of these tools infer, for each ancestral gene tree node, the ancestral species to which the gene belonged to, as well as the event that affected the gene. It is, however, difficult to assess the quality of the reconciliations produced by these approaches, even with the availability of high quality software to simulate gene tree evolution (e.g. SimPhy [44], Asymmetree [45], aevol [46], ZOMBI [47]). A standard benchmarking idea would be to simulate reconciled gene trees and to compare the inferred scenarios with the simulated ones. However, it is not straightforward to perform this comparison. Indeed, reconciled gene trees exhibit three types of valuable information: the tree topology, the gene-species map, and the event labeling. While there exist metrics to measure discrepancies for each of those three criteria individually, we are not aware of any established method to measure disagreements in all three simultaneously. There is a large body of literature on measuring topological differences between trees (e.g. [48–53]). In terms of gene-species mapping discordance, the *path distance* metric [54] applies to gene trees with identical topologies but possibly different species maps, and quantifies how far the species of corresponding nodes are in the gene trees. The metric was mainly introduced to obtain medians in the reconciliation spaces of gene trees. If the gene tree topologies differ, though, the metric cannot be used.

Perhaps the most relevant metric to compare reconciled gene trees is the recent *labeled Robinson-Foulds (RF) distance*, now called ELRF, which accounts for differences in topology and event labeling. Given two gene trees, the distance is the minimum number of edge contractions, edge expansions, and node label substitutions required to transform one gene tree into the other [55]. It is unknown whether this distance can be computed in polynomial time, the main difficulty being that edge operations must have the same label on both endpoints. The authors then proposed a variant of this metric, called LRF, in which edge contractions/expansions are replaced with node insertions/deletions, which can be computed in linear time [56]. Although these are perhaps the only approaches specifically tailored for event-labeled gene tree comparison, their usage has some disadvantages. First, these distances do not take gene-species maps into consideration. Second, the metric suffers from the same well-known shortcomings as the RF distance, see [57] for a discussion on this (for instance, a single misplaced leaf can increase the distance dramatically). Another subtle yet important aspect is the topological uncertainty that can be present in gene trees. In particular, when ancestral species undergo gene duplication episodes (see e.g. [36, 58]), the corresponding gene trees may contain large duplication subtrees. In this case, there is too little phylogenetic signal to infer the topology of such duplication subtrees accurately. However, most approaches penalize discrepancies in those local parts of the gene trees as in any other part, even though predicting incorrect patterns in speciations should be more heavily penalized than in duplication clusters.

In this work, we introduce a novel approach for comparing reconcilied gene trees that considers all the aforementioned components that play a role in reconciliations: the species tree, the reconcilied gene tree, the labeling of their internal nodes by species and events, as well as duplication clusters. This method effectively circumvents the shortcomings of the RF distance. Given two reconciled gene trees on the same set of genes, our dissimilarity measure establishes a correspondence between the gene tree nodes from both trees and applies a penalty if the matched nodes differ in species or event label. As we demonstrate here, due to constraints inherent in reconciliation models, this approach implicitly penalizes topological disagreements between the gene trees, except when the discordance is solely due to consecutive duplication rounds within the same species.

Our measure also has the advantage of being computable in linear time. We first explore some theoretical properties of our approach and show that it functions as a semi-metric in the space of reconciled gene trees. We demonstrate that if non-binary gene trees are considered, the measure does not necessarily satisfy the triangle inequality, although this remains an open question for binary trees. We also provide initial results on the diameters of the PLR, LRF, and ELRF measures, which are important in practice for normalization.

We then validate and demonstrate the utility of our approach through two experiments using simulated datasets. First, by simulating random reconciliations, we show that RF, LRF, and ELRF tend to overestimate tree differences, leading to disproportionately large distance values, especially when even a single leaf is misplaced, which confirms previous observations. In contrast, our measure effectively captures small, moderate, and large distances between reconciliations. Second, we present a study in which our measure helps select the most plausible hypothesis for sequence-generated data. Assuming the existence of a gold standard, we view the possible rootings of a gene tree as the set of hypotheses, and show that in the majority of cases, our approach clearly discriminates one single gene tree as the best among multiple candidates. This contrasts with other measures, which tend to have several candidates as tied for best. This highlights the potential of PLR for creators of gene tree reconstruction methods, which now have access to clearer evaluation tools for their approaches. These studies establish PLR as the first reconciliation measure with greater variability than RF variants and enhanced sensitivity to differences across all components of evolutionary scenarios.

This article is the journal version of our earlier WABI-ALGO2024 conference paper [59]. It significantly expands upon the original version in several ways: In Sect. "Evaluation of the best tree rootings", we introduce a new case study using simulated scenarios, demonstrating how PLR can effectively identify a narrow set of plausible hypotheses close to a gold standard. We have added Fig. 9 as a larger, non-trivial example to help illustrate the kind of information conveyed by our measure and aid readers in understanding its definition. The full proofs, which were omitted from the conference version, are now included in the main text. Finally, we have addressed all reviewer comments received during the WABI review process.

## Preliminary notions

A *tree* is a connected acyclic graph. Unless stated otherwise, all trees in this paper are rooted. For a tree *T*, we denote by *r*(*T*) the root of *T*, by *V*(*T*) and *E*(*T*) its set of nodes and edges, respectively, and by *L*(*T*) its set of leaves. A non-leaf node is called *internal*. For $u, v \in V(T)$, we write $u \preceq _T v$ if *u* is a *descendant* of *v*, i.e., if *v* is on the path between *r*(*T*) and *u* (we write $u \prec _T v$ if, in addition, $u \ne v$). Then *v* is an *ancestor* of *u*. If $u \ne r(T)$, then the *parent* $p_T(u)$ of *u* is the ancestor *v* of *u* such that $uv \in E(T)$, and *u* is a *child* of *v*. A tree *T* is *binary* if each internal node has two children, and a binary *T* is a *caterpillar* if all internal nodes have at most one child that is an internal node, that is, *T* is a path with leaves attached to its nodes.

For $X \subseteq V(T)$, the *lowest common ancestor* of *X*, denoted ${{\,\textrm{lca}\,}}_T(X)$, is the unique node $v \in V(T)$ that is an ancestor of all nodes in $X$, such that no descendant of *v* is also an ancestor of all nodes in *X*. When $|X| = 2$, we may write ${{\,\textrm{lca}\,}}_T(u, v)$ instead of ${{\,\textrm{lca}\,}}_T(\{u, v\})$. For $v \in V(T)$, we write *T*(*v*) for the subtree of *T* rooted at *v*. Note that *L*(*T*(*v*)) is the set of leaves that descend from *v*, which we call the *clade* of *v*. As a shorthand, we may write $L_T(v)$ to denote the clade of *v*, or *L*(*v*) if *T* is understood. The *distance* between two nodes *u*, *v* in *T* is denoted $dist_T(u, v)$, i.e., the length of the undirected path in *T* between *u* and *v*.

### Species trees and reconciled gene trees.

A *species tree* *S* is a tree which we assume to be binary. A *reconciled gene tree* with *S* is a tuple ${\mathcal {G}}= (G, S, \mu , l)$ where *G* is a tree in which each internal node has at least two children (possibly more), *S* is a species tree, $\mu : V(G) \rightarrow V(S)$ maps nodes of *G* to species in *S*, and $l : V(G) \rightarrow \{dup, spec, extant\}$ is an event labeling. We also have the following requirements:

1. *Leaves are from extant species:* for every leaf $v \in L(G)$, $\mu (v) \in L(S)$ and ${l(v) = extant}$. Moreover, an internal node $w \in V(G) \setminus L(G)$ is not considered extant, and satisfies $l(w) \in \{dup, spec\}$;
2. *Time-consistency:* for any two nodes $u, v \in V(G)$, $u \preceq _G v$ implies $\mu (u) \preceq _S \mu (v)$;
3. *Speciations separate species:* for any node $v \in V(G)$ such that $l(v) = spec$, we have $\mu (v) \in V(S) \setminus L(S)$ and *v* has exactly two children $v_1, v_2$ (not more). Moreover, denoting by $s_1, s_2$ the two children of $\mu (v)$ in *S*, we have that $\mu (v_1) \preceq _S s_1$ and $\mu (v_2) \preceq _S s_2$, or $\mu (v_2) \preceq _S s_1$ and $\mu (v_1) \preceq _S s_2$.

**Fig. 1:**
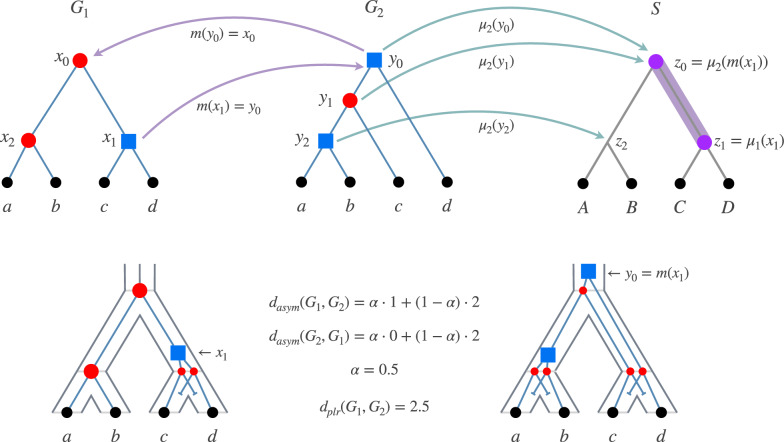
In the upper row, there are two reconciled gene trees ${\mathcal {G}}_1 = (G_1, S, \mu _1, l_1)$ and ${\mathcal {G}}_2 = (G_2, S, \mu _2, l_2)$ as well as a species tree *S*. The event labelings are shown as red circles and blue squares, which represent speciations and duplications, respectively. Lowercase letters *a*, *b*, *c*, *d* depict extant genes, while the corresponding uppercase letters are the species where genes reside. The maps $\mu _1, \mu _2$ use the lca-mapping, that is, $\mu _1(x_0) = z_0, \mu _1(x_1) = z_1, \mu _1(x_2) = z_2$, and ${\mu _2(y_0) = \mu _2(y_1) = z_0}, \mu _2(y_2) = z_2$. The gene trees have the same set of leaves but different topology and event labeling. Purple arrows exemplify the maps $m_{{\mathcal {G}}_1, {\mathcal {G}}_2}(x_1)$, which is the lca of genes *c* and *d*, and $m_{{\mathcal {G}}_2, {\mathcal {G}}_1}(y_0)$, while green arrows illustrate the species map $\mu _2$. The shaded edge in *S* displays the path distance between $\mu _1(x_1) = z_1$ and $\mu _2(m(x_1)) = \mu _2(y_0) = z_0$. The lower row shows the explicit evolution of the gene trees within the species tree. The contribution of $x_1$ to the $d_{path}$ component is 1, because $dist_S( \mu _1(x_1), \mu _2( m(x_1)) ) = 1$, whereas its contribution to $d_{lbl}$ is 0 because $l(x_1) = l(m(x_1)) = dup$. On the other hand, the node $y_0$ from $G_2$ contributes 0 to $d_{path}$ since its correspondent $x_0$ is mapped to the same species, but contributes 1 to $d_{lbl}$ since $l(y_0) = dup$ and $l(x_0) = spec$.

If $\mu$ satisfies $\mu (v) = lca_S( \{\mu (x) : x \in L(v) \})$ for every node $v \in V(G)$, then $\mu$ is called the *lca-mapping* [3, 4]. In this map, all genes map to the lowest possible species according to the rules of reconciliation. These concepts are illustrated in Fig. 1, which presents two reconciled gene trees that use the lca-mapping (see caption). Note that our reconciled gene trees are not restricted to the *lca-mapping*. However, it is known that if $l(v) = spec$, then $\mu (v)$ must indeed be the lowest common ancestor of all the species that appear in the genes below *v* (see e.g. [37, Lemma 2]). However, the converse is not required to hold, that is, a duplication could be mapped to the lowest common ancestral species (or above).

*Isomorphism between reconciled gene trees* Two reconciled gene trees ${\mathcal {G}}_1 = (G_1, S, \mu _1, l_1)$ and ${\mathcal {G}}_2 = (G_2, S, \mu _2, l_2)$ are *isomorphic* if they have the same sets of leaves, use the same species tree, have the same topology, and their corresponding nodes map to the same species and have the same labeling. If this holds, we write ${\mathcal {G}}_1 \simeq {\mathcal {G}}_2$. Formally, ${\mathcal {G}}_1 \simeq {\mathcal {G}}_2$ if there exists a bijection $\phi : V(G_1) \rightarrow V(G_2)$ such that the following holds:

- $L(G_1) = L(G_2)$ and, for each leaf $x \in L(G_1)$, $\phi (x) = x$. In other words, each leaf of $G_1$ is mapped to the same leaf in $G_2$;
- $uv \in E(G_1)$ if and only $\phi (u) \phi (v) \in E(G_2)$;
- for every node $v \in V(G_1)$, $\mu _1(v) = \mu _2( \phi (v) )$ and $l_1(v) = l_2(\phi (v))$.

### The path-label reconciliation (PLR) dissimilarity measure

Let ${\mathcal {G}}_1 = (G_1, S, \mu _1, l_1)$ and ${\mathcal {G}}_2 = (G_2, S, \mu _2, l_2)$ be two reconciled gene trees. We say that ${\mathcal {G}}_1$ and ${\mathcal {G}}_2$ are *comparable* if: (1) they are reconciled with the same species tree *S*; (2) $L(G_1) = L(G_2)$; and (3) for each leaf $x \in L(G_1)$, $\mu _1(x) = \mu _2(x)$ (that is, extant genes map to the same species in both trees). Unless stated otherwise, we assume that all pairs of reconciled trees mentioned are comparable, although (3) could be dropped, see remark below.

For a node $v \in V(G_1)$, we need a corresponding node for *v* in $G_2$. This can be done in multiple ways, and here we assign this corresponding node as the lowest possible node of $G_2$ that is an ancestor of all the descendants of *v*. To put it more formally, define

$$\begin{aligned} m_{{\mathcal {G}}_1, {\mathcal {G}}_2}(v) = lca_{G_2}( L(G_1(v)) ) \end{aligned}$$

which is the lowest common ancestor in $G_2$ of the clade of *v*. Note that this is well-defined since $L(G_1) = L(G_2)$. For instance in Fig. 1, $m_{{\mathcal {G}}_1, {\mathcal {G}}_2}(x_1) = y_0$. When ${\mathcal {G}}_1, {\mathcal {G}}_2$ are clear from the context, we may write *m*(*v*) instead of $m_{{\mathcal {G}}_1,{\mathcal {G}}_2}(v)$. In essence, this is the lca-mapping, but applied between two gene trees. Note that such mappings are usually applied between gene and species trees, but [60] also introduced the ancestral gene-gene map idea (or more specifically, ancestral RNA-gene maps).

Our measure has two components: one for the discrepancies in the species mappings, and one for the labelings. These components are defined as:

$$\begin{aligned} d_{path}({\mathcal {G}}_1, {\mathcal {G}}_2)&= \sum _{v \in V(G_1)} dist_S( \mu _1(v), \mu _2( m(v)) ) \\ d_{lbl}({\mathcal {G}}_1, {\mathcal {G}}_2)&= | \{ v \in V(G_1) : l_1(v) \ne l_2(m(v)) \} | \end{aligned}$$

In words, in $d_{path}$, each term $dist_S(\mu _1(v), \mu _2(m(v)))$ penalizes *v* by how far its species is from the species of its correspondent *m*(*v*), and $d_{lbl}$ is simply the number of nodes of $G_1$ whose label differ from their correspondent in $G_2$.

We assume the existence of a given parameter $\alpha \in [0, 1]$ to weigh these components, and define the *asymmetric dissimilarity* between ${\mathcal {G}}_1$ and ${\mathcal {G}}_2$ as:

$$\begin{aligned} d_{asym}({\mathcal {G}}_1, {\mathcal {G}}_2) = \alpha \cdot d_{path}({\mathcal {G}}_1, {\mathcal {G}}_2) + (1 - \alpha ) \cdot d_{lbl}({\mathcal {G}}_1, {\mathcal {G}}_2). \end{aligned}$$

Note that when $\alpha = 1$ and $G_1, G_2$ have the same topology, then $d_{asym}$ is exactly the *path distance metric* studied in [54]. Our dissimilarity measure generalizes this by allowing trees with different topologies and by considering node labels. One could ignore the $\alpha$ parameter by weighing $d_{path}$ and $d_{lbl}$ equally, which can be achieved with $\alpha = 0.5$. Also notice that $d_{path}$ may be adapted to species trees with branch lengths.

It is easy to see that $d_{asym}$ is not symmetric. For instance, suppose that ${\mathcal {G}}_1$ consists of a binary gene tree with several internal nodes mapping to different species, and ${\mathcal {G}}_2$ consists of a star tree with a single internal node, such that both roots are duplications that map to the same species. Then $d_{asym}({\mathcal {G}}_1, {\mathcal {G}}_2)$ can be proportional to the number of internal nodes of $G_1$, whereas $d_{asym}({\mathcal {G}}_2, {\mathcal {G}}_1) = 0$.

The Path-Label Reconciliation (PLR) dissimilarity is therefore defined as

$$\begin{aligned} d_{plr}({\mathcal {G}}_1, {\mathcal {G}}_2) = d_{asym}({\mathcal {G}}_1, {\mathcal {G}}_2) + d_{asym}({\mathcal {G}}_2, {\mathcal {G}}_1) \end{aligned}$$

If ${\mathcal {G}}_1$ and ${\mathcal {G}}_2$ are not comparable, then we define $d_{plr}({\mathcal {G}}_1, {\mathcal {G}}_2) = \infty$.

In Fig. 1 we exemplify all the components of the dissimilarity measure. In the example, following the $\mu _1, \mu _2$ maps given in the caption, if we count the respective costs of $x_0, x_1, x_2$, we have $d_{path}({\mathcal {G}}_1, {\mathcal {G}}_2) = 0 + 1 + 0 = 1$ and $d_{lbl}({\mathcal {G}}_1, {\mathcal {G}}_2) = 1 + 0 + 1 = 2$. If we put $\alpha = 0.5$, we get $d_{asym}({\mathcal {G}}_1, {\mathcal {G}}_2) = 0.5 \cdot 1 + 0.5 \cdot 2 = 1.5$. As for the costs of $y_0, y_1, y_2$, we get $d_{path}({\mathcal {G}}_2, {\mathcal {G}}_1) = 0 + 0 + 0$ and $d_{lbl}({\mathcal {G}}_2, {\mathcal {G}}_1) = 1 + 0 + 1 = 2$, and thus $d_{asym}({\mathcal {G}}_2, {\mathcal {G}}_1) = 1$. Therefore, $d_{plr}({\mathcal {G}}_1, {\mathcal {G}}_2) = 2.5$.

*A remark on leaves belonging to the same species* Recall that condition (3) of comparability requires $\mu _1(x) = \mu _2(x)$ for every leaf $x \in L(G_1)$. Although this assumption usually follows from the knowledge of the species of a gene, it may not hold in some contexts. Indeed, in metagenomics even the species of extant genes is unknown and needs to be inferred (see for example [58]). Therefore, for an extant gene *x*, two different reconciliation algorithms may predict that *x* belongs to a different species, leading to $\mu _1(x) \ne \mu _2(x)$. Although condition (3) is useful in the proofs that follow, we note that it is not required in the definition of $d_{plr}$, and the latter remains well-defined even if we drop this condition. Note that with the current definition, the contribution of the leaves to the path-distance component of our metric is zero. By droppping this condition, leaves could have a non-zero contribution to this component. Therefore, $d_{plr}$ could be used to also compare gene trees with predicted gene-species maps that differ even at the level of leaves (although the theory developed hereafter may need revision for this case).

*A remark on setting* $\alpha$ The reader may notice that if $\alpha$ is ignored in $d_{plr}$, or set to a constant, the $d_{path}$ component can easily outweigh the $d_{lbl}$ component. This is because in the worst case, $d_{path}({\mathcal {G}}_1, {\mathcal {G}}_2)$ can be in $\Theta (nm)$, where *n* is the number of species leaves and *m* is the number of gene tree leaves, which occurs if most nodes of ${\mathcal {G}}_1$ are mapped to nodes of ${\mathcal {G}}_2$ with $\Theta (n)$ path distance in *S* (see the diameter section for a detailed analysis). On the other hand, the $d_{lbl}({\mathcal {G}}_1, {\mathcal {G}}_2)$ component is always *O*(*m*), as it only depends on the number of nodes in the gene tree. This quadratic-versus-linear effect can be prevented by making $\alpha$ depend on *n*. For instance, one may put $\alpha = 1/n$, or more generally $\alpha = c/n$ for some constant *c*.

*A remark on scenarios with horizontal transfer events* In the presence of horizontal gene transfers, gene tree nodes can also undergo a *transfer* event, and a different notion of time-consistency than ours is typically used (see e.g. [61]). Nonetheless, such reconciliations also include a gene-species map $\mu$ and a labeling function *l*, and $d_{plr}$ is also well-defined in this context. On the other hand, it is unclear whether path distances are appropriate to compare transferred genes, and again, the theory that follows may need to be adapted to allow transfers.

***Least duplication-resolved gene trees*** Consider a reconciled gene tree ${\mathcal {G}}= (G, S, \mu , l)$. If, in *G*, there is a connected subtree consisting only of duplication nodes, all mapped to the same species, then it is difficult to postulate on the exact topology of the duplication subtree due to the lack of clear phylogenetic signals. One solution is to contract the subtree into a single node to model the uncertainty. Contracting weakly supported branches in gene trees can be useful to detect and correct errors in dubious duplication nodes [62]. Moreover, special cases of least-duplication resolved trees such as discriminating co-trees arise in the context of orthology detection [63, 64]. To this end, we say that an edge $uv \in E(G)$ is *redundant* if $\mu (u) = \mu (v)$ and $l(u) = l(v) = dup$. We then say that ${\mathcal {G}}$ is *least duplication-resolved* if no edge *uv* of *G* is redundant.

**Fig. 2:**
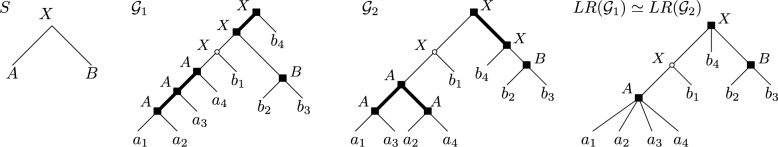
Two different reconciled gene trees ${\mathcal {G}}_1, {\mathcal {G}}_2$, where redundant edges are bold (again, lowercase letters indicate the species). Their $d_{plr}$ value is 0, since one can check that all duplications in species $W \in \{A, X, B\}$ in either tree maps to a duplication in the same *W* in the other tree, and the *X* speciation to an *X* speciation. On the right, the least duplication-resolved version of the trees, showing that ${\mathcal {G}}_1 \simeq _d {\mathcal {G}}_2$.

Suppose that ${\mathcal {G}}$ is *not* least duplication-resolved, and let $uv \in E(G)$ be a redundant edge, with $u = p_G(v)$. We denote by ${\mathcal {G}}/ uv$ the reconciled gene tree obtained by contracting *uv* in *G* and updating $\mu$ and *l* accordingly. More specifically, ${\mathcal {G}}/ uv = (G', S, \mu ', l')$, where: $G'$ is obtained from *G* by deleting *v* and its incident edges and, for each child $v'$ of *v* in *G*, adding the edge $uv'$; and then putting $\mu '(w) = \mu (w)$ and $l'(w) = l(w)$ for every $w \in V(G')$. If $R \subseteq E(G)$ is a set of redundant edges of ${\mathcal {G}}$, then ${\mathcal {G}}/ R$ is the reconciled gene tree obtained after contracting every edge in *R*, in any order. If *R* is the set of all redundant edges of ${\mathcal {G}}$, then we define $LR({\mathcal {G}}) = {\mathcal {G}}/ R$, called the *least duplication-resolved subtree* of ${\mathcal {G}}$. It is not difficult to see that such a subtree is unique, least duplication-resolved, and satisfies all conditions of a reconciled gene tree. Figure 2 shows two gene trees and their least duplication-resolved version (note that two consecutive duplications in distinct species remain).

For two reconciled gene trees ${\mathcal {G}}_1, {\mathcal {G}}_2$, we write ${\mathcal {G}}_1 \simeq _d {\mathcal {G}}_2$ if $LR({\mathcal {G}}_1) \simeq LR({\mathcal {G}}_2)$. This means that ${\mathcal {G}}_1$ and ${\mathcal {G}}_2$ may differ, but every form of disagreement is due to redundant edges, and they become identical in their least duplication-resolved form. The following will be useful to analyze these trees.

#### Lemma 1

Let ${\mathcal {G}}= (G, S, \mu , l)$ be a reconciled gene tree that is least duplication-resolved. Let $u,v \in V(G)$ be such that $v \prec _G u$. Then either $\mu (u) \ne \mu (v)$ or $l(u) \ne l(v)$.

#### Proof

Let $u = u_1, u_2, \ldots , u_k = v$ be the path from *u* to *v* in *G*. Suppose that there is a speciation on the path, that is, there is some $i \in \{1, 2, \ldots , k - 1\}$ such that $l(u_i) = spec$. By the *speciations separate species* requirement, denoting $s = \mu (u_i)$ and letting $s_1, s_2$ be the children of *s* in *S*, we have $\mu (u_{i+1}) \preceq s_1$ or $\mu (u_{i+1}) \preceq s_2$. Either way, $\mu (u_{i+1}) \prec \mu (u_i)$. By the *time-consistency* requirement, we then have

$$\begin{aligned} \mu (v) = \mu (u_k) \preceq \mu (u_{k-1}) \preceq \ldots \preceq \mu (u_{i+1}) \prec \mu (u_i) \preceq \mu (u_{i-1}) \preceq \ldots \preceq \mu (u_1) = \mu (u). \end{aligned}$$

The presence of a $\prec$ in this chain implies $\mu (v) \ne \mu (u)$, as desired.

So suppose that $l(u_1) = \ldots = l(u_{k-1}) = dup$. If $l(v) \ne dup$, we are done, so assume $l(v) = l(u_k) = dup$. By the definition of duplication least-resolved, we must have $\mu (u_k) \ne \mu (u_{k-1})$. By time-consistency, this implies $\mu (v) = \mu (u_k) \prec \mu (u_{k-1}) \preceq \mu (u_1) = \mu (u)$ and we are done. $\square$

## Properties of the path-label reconciliation (PLR) dissimilarity

We first show that in terms of time complexity, $d_{plr}({\mathcal {G}}_1, {\mathcal {G}}_2)$ can be computed in linear time, using appropriate data structures, in a very straightforward manner as shown in Algorithm 1. The details of a linear-time implementation can be found in the proof of Theorem 1.

**Algorithm 1:**
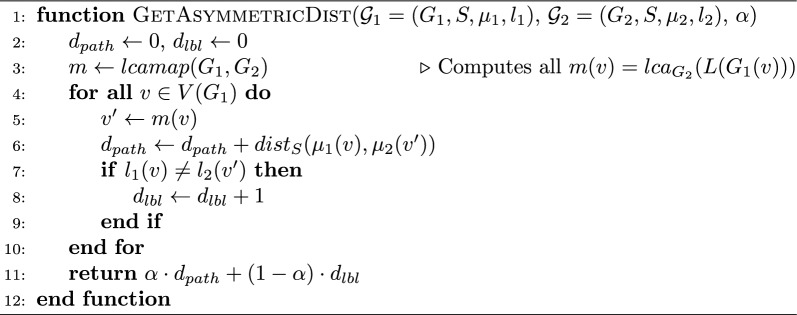
Computing $d_{asym}$ in one direction.

### Theorem 1

The value $d_{plr}({\mathcal {G}}_1, {\mathcal {G}}_2)$ can be computed in time $O(|V(G_1)| + |V(G_2)| + |V(S)|)$.

### Proof

We argue that Algorithm 1 can be implemented to run in time $O(|V(G_1)| + |V(G_2)| + |V(S)|)$, which clearly proves the statement since we only need to run it twice (once for ${\mathcal {G}}_1$ versus ${\mathcal {G}}_2$, and once for ${\mathcal {G}}_2$ versus ${\mathcal {G}}_1$). We assume that $G_1$, $G_2$, and *S* are pre-processed to answer lowest common ancestor queries between any two nodes in constant time. This pre-processing time is linear for each tree [65], and therefore this step takes time $O(|V(G_1)| + |V(G_2)| + |V(S)|)$. We also assume that we know the depth of each node *x* of *S*, denoted *depth*(*x*), which is the distance between *x* and the root. This can easily be computed by a linear-time preorder traversal of *S*. It is not difficult to compute $m = lcamap(G_1, G_2)$ in time $O(|V(G_1)| + |V(G_2)|)$ using the *lca* pre-processing and dynamic programming. Indeed, for a gene tree node $v \in V(G_1)$ with children $v_1, \ldots , v_h$, we have $m(v) = lca_{G_2}( \{m(v_1), \ldots , m(v_h) \})$. The latter *lca* expression can be computed with $h - 1$ *lca* queries as follows. Define $w_{1, i} = lca_{G_2}( \{m(v_1), \ldots , m(v_i)\} )$. First compute $w_{1,2} = lca_{G_2}(m(v_1), m(v_2))$, then $w_{1,3} = lca_{G_2}(w_{12}, m(v_3))$, and so on until $m(v) = w_{1, h} = lca_{G_2}(w_{1,h-1}, m(v_l))$, each in *O*(1) time. Since *h* is the number of edges between *v* and its children, the number of *lca* queries required throughout the execution of the whole algorithm is less than the number of edges of $G_1$, which is $O(|V(G_1)|)$.

Then consider the main loop of the algorithm. For each $v \in V(G_1)$, we can obtain $dist_S(\mu _1(v), \mu _2(v'))$ in constant time, since it is equal to $depth(\mu _1(v)) + depth(\mu _2(v')) - 2 \cdot depth( lca_S(\mu _1(v), \mu _2(v') ) )$. It follows that each $v \in V(G_1)$ can be dealt with in *O*(1) time and the loop of the algorithm takes time $O(|V(G_1)|)$, which does not add to the complexity. $\square$

### A semi-metric under least duplication-resolved equivalence

Let us recall the mathematical notion of a metric, which can be defined as a triple $(X, d, \equiv )$ where *X* is a set, $d : X \times X \rightarrow {\mathbb {R}}$ is a dissimilarity function, and $\equiv$ is a binary equality operator on *X* (that is, for $x, y \in X$, $x \equiv y$ is *true* if *x* and *y* are considered equal, and otherwise $x \equiv y$ is *false*, which we may write as $\not \equiv$). Moreover, the following conditions are satisfied:

- (identity) for all $x \in X$, $d(x, x) = 0$;
- (positivity) for all $x, y \in X$, if $x \not \equiv y$, then $d(x, y)> 0$;
- (symmetry) for all $x, y \in X$, $d(x, y) = d(y, x)$;
- (triangle inequality) for all $x, y, z \in X$, $d(x, z) \le d(x, y) + d(y, z)$.

If all the above conditions are satisfied, except the triangle inequality, then $(X, d, \equiv )$ is a *semi-metric*. If *X* is clear from the context, we call $d$ a *metric (or semi-metric) under* $\equiv$.

In our case, we consider the set of all reconciled gene trees, with $d_{plr}$ as our dissimilarity function. As for the equality operator, we may consider $\simeq$ or $\simeq _d$. In general, $d_{plr}$ does not always meet the *positivity* requirement under $\simeq$. That is, ${\mathcal {G}}_1 \not \simeq {\mathcal {G}}_2$ does not necessarily imply $d_{plr}({\mathcal {G}}_1, {\mathcal {G}}_2)> 0$. Consider for example two gene trees with different topologies, but whose internal nodes are all duplications in the same species (in which case all internal nodes incur a path and label penalty of 0). For a more elaborate example, see Fig. 2.

However, we can show that $d_{plr}$ is a semi-metric under $\simeq _d$. The most difficult part is to show that ${\mathcal {G}}_1 \not \simeq _d {\mathcal {G}}_2$ implies $d_{plr}({\mathcal {G}}_1, {\mathcal {G}}_2)> 0$. We first need to show that contracting the trees to their least duplication-resolved form cannot increase the dissimilarity.

#### Lemma 2

Let ${\mathcal {G}}_1 = (G_1, S, \mu _1, l_1), {\mathcal {G}}_2 = (G_2, S, \mu _2, l_2)$ be comparable reconciled gene trees, and let $uv \in E(G_1)$ be a redundant edge. Then $d_{plr}({\mathcal {G}}_1, {\mathcal {G}}_2) \ge d_{plr}({\mathcal {G}}_1 / uv, {\mathcal {G}}_2)$.

#### Proof

Denote ${\mathcal {G}}_1 / uv = {\mathcal {G}}'_1 = (G'_1, S, \mu '_1, l'_1)$, and note that $V(G'_1) = V(G_1) \setminus \{v\}$. Also recall that in a contraction, the parent subsumes the child, and so contractions do not alter the set of descendants of a node. Thus $L_{G_1}(x) = L_{G'_1}(x)$ for all $x \in V(G'_1)$. Therefore, for $w \in V(G_1) \setminus \{v\}$, we have $m_{{\mathcal {G}}_1, {\mathcal {G}}_2}(w) = m_{{\mathcal {G}}'_1, {\mathcal {G}}_2}(w)$. Since *w* and its correspondent $m_{{\mathcal {G}}_1, {\mathcal {G}}_2}(w)$ both have the same species map and label before and after the contraction, the contribution of *w* to $d_{path}$ and $d_{lbl}$ is the same in either ${\mathcal {G}}_1$ and ${\mathcal {G}}'_1$. As this holds for every *w* that is still in $G'_1$, we get $d_{asym}({\mathcal {G}}_1, {\mathcal {G}}_2) \ge d_{asym}({\mathcal {G}}'_1, {\mathcal {G}}_2)$.

Now let $w \in V(G_2)$. If $m_{{\mathcal {G}}_2, {\mathcal {G}}_1}(w) \ne v$, then $m_{{\mathcal {G}}_2, {\mathcal {G}}'_1}(w) = m_{{\mathcal {G}}_2, {\mathcal {G}}_1}(w)$, since $m_{{\mathcal {G}}_2, {\mathcal {G}}_1}(w)$ is still a common ancestor of *L*(*w*) in $G'_1$, and no such lower ancestor can exist as it would also exist in $G_1$. The contribution of *w* to $d_{path}$ and $d_{lbl}$ is therefore unchanged. Suppose instead that $m_{{\mathcal {G}}_2, {\mathcal {G}}_1}(w) = v$. Then in $G'_1$, *u* is a common ancestor of $L_{G_2}(w)$, and no such lower ancestor could exist, as it would also be in $G_1$. In other words, $m_{{\mathcal {G}}_2, {\mathcal {G}}'_1}(w) = u$. Since *uv* is redundant, $\mu '_1(u) = \mu _1(u) = \mu _1(v)$ and $l'_1(u) = l_1(u) = l_1(v)$. As the contribution of *w* to $d_{path}$ and $d_{lbl}$ is based on $\mu _1(v)$ and $l_1(v)$, it is unchanged in ${\mathcal {G}}'_1$, and so $d_{asym}({\mathcal {G}}_2, {\mathcal {G}}_1) = d_{asym}({\mathcal {G}}_2, {\mathcal {G}}'_1)$, which concludes the proof. $\square$

Since Lemma 2 can be applied to any sequence of contractions, in either ${\mathcal {G}}_1$ or ${\mathcal {G}}_2$ by symmetry, we get the following.

#### Corollary 1

Let ${\mathcal {G}}_1 = (G_1, S, \mu _1, l_1), {\mathcal {G}}_2 = (G_2, S, \mu _2, l_2)$ be comparable reconciled gene trees. Then $d_{plr}({\mathcal {G}}_1, {\mathcal {G}}_2) \ge d_{plr}(LR({\mathcal {G}}_1), LR({\mathcal {G}}_2))$.

The above is sufficient to deduce that if $\simeq _d$ is interpreted as “being the same reconciled tree”, then we have a semi-metric, unless $\alpha = 0$ or $\alpha = 1$.

#### Theorem 2

For any $\alpha \in (0, 1)$, $d_{plr}$ is a semi-metric under $\simeq _d$.

#### Proof

*Identity.* Let ${\mathcal {G}}= (G, S, \mu , l)$ be a reconciled gene tree. Let us argue that $d({\mathcal {G}}, {\mathcal {G}}) = 0$. Let $v \in V(G)$, and notice that $m_{{\mathcal {G}}, {\mathcal {G}}}(v) = v$. Therefore, the distance in *S* between $\mu (v)$ and $\mu (m(v))$ is 0 and *v* incurs no label penalty. Since this holds for every *v*, the dissimilarity between ${\mathcal {G}}$ and ${\mathcal {G}}$ is 0.

*Symmetry.* Observe that $d_{plr}$ is symmetric by design, as it adds both terms $d_{asym}({\mathcal {G}}_1, {\mathcal {G}}_2)$ and $d_{asym}({\mathcal {G}}_2, {\mathcal {G}}_1)$ whether we calculate $d_{plr}({\mathcal {G}}_1, {\mathcal {G}}_2)$ or $d_{plr}({\mathcal {G}}_2, {\mathcal {G}}_1)$.

*Positivity.* The rest of the proof is dedicated to showing that the positivity requirement is met under $\simeq _d$. Let ${\mathcal {G}}_1 = (G_1, S, \mu _1, l_1)$ and ${\mathcal {G}}_2 = (G_2, S, \mu _2, l_2)$ such that ${\mathcal {G}}_1 \not \simeq _d {\mathcal {G}}_2$. We need to show that $d_{plr}({\mathcal {G}}_1, {\mathcal {G}}_2)> 0$. We may assume that ${\mathcal {G}}_1, {\mathcal {G}}_2$ are least duplication-resolved. This is because if not, then by Corollary 1, $d_{plr}({\mathcal {G}}_1, {\mathcal {G}}_2) \ge d_{plr}(LR({\mathcal {G}}_1), LR({\mathcal {G}}_2))$, so if we prove positivity for any pair of least duplication-resolved gene trees, it will also hold for any pair of trees.

So, from now on we assume that ${\mathcal {G}}_1$ and ${\mathcal {G}}_2$ are least duplication-resolved, and that ${\mathcal {G}}_1 \not \simeq {\mathcal {G}}_2$ (we may replace $\not \simeq _d$ with $\not \simeq$ now, since the two notions coincide on least duplication-resolved trees). To ease notation, we use $m_{12}$ instead of $m_{{\mathcal {G}}_1, {\mathcal {G}}_2}$ and $m_{21}$ instead of $m_{{\mathcal {G}}_2, {\mathcal {G}}_1}$. To ease further, for $v \in V(G_1)$, we may denote $v' = m_{12}(v)$ for the correspondent of *v* in $G_2$.

Suppose first that for every $v \in V(G_1)$, $L(v) = L(m_{12}(v))$ *and* that for every $w \in V(G_2)$, $L(w) = L(m_{21}(w))$. This means that both trees have exactly the same set of clades. Therefore, $G_1$ and $G_2$ are isomorphic, i.e., they are the same tree (if we ignore the gene-species maps and event labels). Since for all $v \in V(G_1)$, $L(v) = L(m_{12}(v))$, it is easy to see that $uv \in E(G_1)$ if and only if $m_{12}(u) m_{12}(v) \in E(G_2)$. This means that if $\mu _1(v) = \mu _2(m_{12}(v))$ and $l_1(v) = l_2(m_{12}(v))$ for every $v \in V(G_1)$, then $m_{12}$ meets all the requirements to deduce that ${\mathcal {G}}_1 \simeq {\mathcal {G}}_2$, which we assume is not the case. Therefore, there must be some $v \in V(G_1)$ such that $\mu _1(v) \ne \mu _2(v')$ or $l_1(v) \ne l_2(v')$, and then $d_{plr}({\mathcal {G}}_1, {\mathcal {G}}_2)> 0$ as desired.

So, we may assume that there is some $v \in V(G_1)$ such that $L(v) \ne L(m_{12}(v))$, or some $w \in V(G_2)$ such that $L(w) \ne L(m_{21}(w))$. We may assume that the former occurs—which is without loss of generality since we can swap the roles of ${\mathcal {G}}_1$ and ${\mathcal {G}}_2$ as $d_{plr}$ is symmetric.

Let $v \in V(G_1)$ such that $L(v) \ne L(v')$. Because $v'$ is the lowest common ancestor of *L*(*v*) in $G_2$, it must be that *L*(*v*) is a strict subset of $L(v')$. If *v* and $v'$ have different species map or label, the dissimilarity will be non-zero and we are done, so assume that $\mu _1(v) = \mu _2(v')$ and $l_1(v) = l_2(v')$.

Let $v'' = m_{21}(v')$ be the node of $G_1$ that corresponds to $v'$. We have $L(v) \subset L(v') \subseteq L(v'')$, and so $v''$ must be a strict ancestor of *v*. Since ${\mathcal {G}}_1$ and ${\mathcal {G}}_2$ are least duplication-resolved, by Lemma 1, $v''$ either has a different species or a different label than *v*, and thus different from $v'$ as well. Since $v'$ has a difference with its correspondent $v''$, the dissimilarity is non-zero. Having handled every case, it follows that ${\mathcal {G}}_1$ and ${\mathcal {G}}_2$ have non-zero dissimilarity. $\square$

We next show that, despite being a semi-metric, the $d_{plr}$ dissimilarity measure is not a metric since it does not satisfy the triangle inequality on non-binary gene trees, regardless of $\alpha$. If $\alpha$ is a constant, it can even be far from satisfying the inequality.

**Fig. 3:**
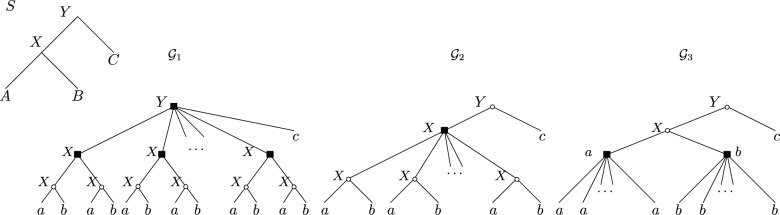
A species tree *S* and reconciled gene trees ${\mathcal {G}}_1, {\mathcal {G}}_2, {\mathcal {G}}_3$ that violate the triangle inequality.

#### Proposition 1

For any $\alpha \in [0, 1]$, possibly depending on the number of leaves of the gene trees, $d_{plr}$ does not necessarily satisfy the triangle inequality. This is true even if the gene trees use the lca-mapping and are least-resolved.

Moreover, for any fixed $\alpha < 1$, the quantity $d_{plr}({\mathcal {G}}_1, {\mathcal {G}}_3)$ can be arbitrarily larger than $d_{plr}({\mathcal {G}}_1, {\mathcal {G}}_2) + d_{plr}({\mathcal {G}}_2, {\mathcal {G}}_3)$.

#### Proof

Consider the tree reconciled gene trees illustrated in Fig. 3. Suppose that the root of the ${\mathcal {G}}_1$ gene tree has, as children, $k \ge 2$ copies of an ((*a*, *b*), (*a*, *b*)) subtree (here, we use *a*, *b*, *c* to denote genes in species *A*, *B*, *C*, respectively, with the understanding that every leaf can be distinguished). Then the gene tree of ${\mathcal {G}}_2$ has 2*k* copies of an (*a*, *b*) subtree. Assume that every (*a*, *b*) subtree of ${\mathcal {G}}_1$ maps uniquely to some (*a*, *b*) subtree of ${\mathcal {G}}_2$. One can check that the three reconciled gene trees use the lca-mapping.

We now consider the asymmetric dissimilarity between each pair of reconciled gene trees.

- $d_{asym}({\mathcal {G}}_1, {\mathcal {G}}_2) = 1 - \alpha$. The speciations in *X* of ${\mathcal {G}}_1$ each map to a speciation in *X* in ${\mathcal {G}}_2$, so they incur no cost. Each duplication in *X* of ${\mathcal {G}}_1$ must map to the duplication in *X* of ${\mathcal {G}}_2$, again at no cost. Only the duplication mapped to *Y* maps to a speciation mapped to *Y*, incurring a cost of $1 - \alpha$.
- $d_{asym}({\mathcal {G}}_2, {\mathcal {G}}_1) = 1$. The root of ${\mathcal {G}}_2$ is a speciation in *Y* that maps to a duplication in *Y* (cost $(1 - \alpha )$ for the label change) and the duplication in *X* maps to the root of ${\mathcal {G}}_1$ (mapped to *Y*, cost of $\alpha$ for the path of distance 1). The (*a*, *b*) subtrees incur no cost.
- $d_{asym}({\mathcal {G}}_2, {\mathcal {G}}_3) = 1 - \alpha$. Each (*a*, *b*) subtree in ${\mathcal {G}}_2$ maps to the speciation in *X* of ${\mathcal {G}}_3$, so no cost incurred. The only cost incurred is $1 - \alpha$ for the duplication in *X* of ${\mathcal {G}}_2$ mapped to a speciation in *X*.
- $d_{asym}({\mathcal {G}}_3, {\mathcal {G}}_2) = 1 + \alpha$. The duplications in *a* and *b* map to a duplication in *X* (cost $2\alpha$) and the speciation in *X* to a duplication in *X* (cost $1 - \alpha$).

From the above, it follows that $d_{plr}({\mathcal {G}}_1, {\mathcal {G}}_2) + d_{plr}({\mathcal {G}}_2, {\mathcal {G}}_3) = 4 - \alpha$.

Let us now compare ${\mathcal {G}}_1$ and ${\mathcal {G}}_3$:

- $d_{asym}({\mathcal {G}}_1, {\mathcal {G}}_3) = k(1 - \alpha ) + 1 - \alpha$. The (*a*, *b*) subtrees of ${\mathcal {G}}_1$ incur no cost. However, each duplication in *X* maps to the speciation in *X* of ${\mathcal {G}}_3$, and there are *k* of them, for a cost of $k(1 - \alpha )$. The *Y* duplication also pays $(1 - \alpha )$.
- $d_{asym}({\mathcal {G}}_3, {\mathcal {G}}_1) = 2 + 3\alpha$. Every internal node of ${\mathcal {G}}_3$ maps to the root of ${\mathcal {G}}_1$. The *a* and *b* duplications of ${\mathcal {G}}_3$ each map to a duplication in *Y* of ${\mathcal {G}}_1$ for a cost of $2 \cdot 2\alpha$. The speciation in *X* costs $\alpha + 1 - \alpha = 1$ and the speciation in *Y* costs $1 - \alpha$.

By summing the two directions and simplifying, we get $d_{plr}({\mathcal {G}}_1, {\mathcal {G}}_3) = k(1 - \alpha ) + 2\alpha + 3$.

Observe that if $\alpha < 1$ is a fixed constant, the quantity $k(1 - \alpha ) + 2\alpha + 3$ can be made arbitrarily large as *k* grows, whereas $d_{plr}({\mathcal {G}}_1, {\mathcal {G}}_2) + d_{plr}({\mathcal {G}}_2, {\mathcal {G}}_3) = 4 - \alpha$ is a constant. This justifies the second part of the proposition.

If $\alpha$ is not necessarily fixed, then assuming $k \ge 2$ and any $\alpha \le 1$,

$$\begin{aligned} k(1 - \alpha ) + 2\alpha + 3 \ge 2 \cdot (1 - \alpha ) + 2 \alpha + 3 = 5> 4 - \alpha \end{aligned}$$

which establishes the lack of a triangle inequality. $\square$

We observe that in the example from Fig. 3, the triangle inequality is violated mainly because the trees are heavily imbalanced in terms of number of internal nodes. We could not find counter-examples in which all trees are *binary*.

## Diameters

We now study the question of computing the *diameter* of $d_{plr}$, which is the maximum possible dissimilarity achievable over a given instance size. This can be useful in practice for normalization, since we can compare heterogeneous datasets by dividing obtained dissimilarities by the diameter. In the context of general trees, the diameter is usually the maximum dissimilarity among all pairs of trees with the same number of leaves *n*. In reconciled gene trees though, there are multiple ways to define the diameter. We may fix two numbers *n*, *m*, and find the maximum $d_{plr}$ value among all species trees on *n* leaves and pairs of gene trees on *m* leaves. Or, we could decide to fix the species tree *S*, and find the gene trees over *m* leaves of maximum $d_{plr}$ value with respect to *S*. Or, we could fix the species tree *S*, and for each species leaf $s \in L(S)$ also fix the number $m_s$ of extant genes that belong to *s*, and find the most distant gene trees under these parameters.

Even the simplest forms of diameters are not trivial to determine. We thus provide initial results by determining the diameter in the case that the species tree *S* is fixed, and gene trees contain exactly one gene per species. Even though this assumption may not hold in practice, we hope that the bounds established here can be extended to broader classes of scenarios in the future. We leave the question of finding the theoretical values of the other diameters as open problems.

For a fixed species tree *S*, let ${\textsf{G}}^S$ represent the set of all reconciled gene trees ${\mathcal {G}}= (G, S, \mu , l)$, such that for each $s \in L(S)$, exactly one leaf *x* of *G* satisfies $\mu (x) = s$. Since each leaf of *G* is uniquely identifiable by its species, we assume that all the elements of ${\textsf{G}}^S$ have the same leaves and are pairwise-comparable.

We define the *diameter for fixed S* as:

$$\begin{aligned} diam(d_{plr},S) = \max \limits _{{\mathcal {G}}_1,{\mathcal {G}}_2 \in {\textsf{G}}^S} \Big \{ d_{plr}({\mathcal {G}}_1,{\mathcal {G}}_2) \Big \}. \end{aligned}$$

In terms of $d_{lbl}$, in the worst case $d_{lbl}({\mathcal {G}}_1, {\mathcal {G}}_2)$ is the number of internal nodes of the gene tree of ${\mathcal {G}}_1$, which occurs when all labels differ. We next characterize the maximum possible path distance. It is tempting to make every node of ${\mathcal {G}}_1$ map to a deepest leaf of *S*, and every node of ${\mathcal {G}}_2$ to the root of *S*, thereby maximizing $dist_S(\mu _1(v), \mu _2(m(v)))$ for every node *v*, but such an example may not satisfy the rules of reconciliation.

For a species tree *S*, let

$$\begin{aligned} H(S) = \sum \limits _{x \in V(S)\setminus L(S)} dist_S(x,r(S)) \end{aligned}$$

be the sum of root-to-internal node distances.

### Lemma 3

Let *S* be a species tree on $n \ge 1$ leaves. Let ${\mathcal {G}}_1$ and ${\mathcal {G}}_2$ be two reconciled trees in ${\textsf{G}}^S$. Then $d_{path}({\mathcal {G}}_1,{\mathcal {G}}_2) \le H(S) \le (n - 1)(n - 2)/2$.

### Proof

Let us focus on $d_{path}({\mathcal {G}}_1, {\mathcal {G}}_2) \le H(S)$. Let ${\mathcal {G}}_1 = (G_1, S, \mu _1, l_1)$ and ${\mathcal {G}}_2 = (G_2, S, \mu _2, l_2)$. For the duration of the proof, let $\lambda : V(G_1) \rightarrow V(S)$ be the lca-mapping between $G_1$ and *S*. Recall that $\lambda (v) = lca_S( \{ \mu _1(l) : l \in L(v) \} )$ is the lowest common ancestor of all the species that appear below *v*. Also recall that $\mu _1(v) \succeq _S \lambda (v)$ for every $v \in V(G_1)$, since by time-consistency, $\mu _1(v)$ must be an ancestor of $\mu _1(l)$ for every $l \in L(v)$.

Let $v \in V(G_1) \setminus L(G_1)$ and denote $v' = m_{{\mathcal {G}}_1, {\mathcal {G}}_2}(v)$. We claim that

$$\begin{aligned}dist_S( \mu _1(v), \mu _2(v') ) \le dist_S( \lambda (v), r(S) ). \end{aligned}$$

As mentioned, we have that $\mu _1(v) \succeq _S \lambda (v)$. Moreover, by the definition of $m_{{\mathcal {G}}_1, {\mathcal {G}}_2}$, $L(v')$ contains all the leaves in *L*(*v*), so we also deduce that $\mu _2(v')$ is an ancestor of $\mu _2(l) = \mu _1(l)$ for every leaf $l \in L_{G_1}(v)$ (since we assume that ${\mathcal {G}}_1$ and ${\mathcal {G}}_2$ are comparable). Therefore, $\mu _2(v') \succeq _S \lambda (v)$ as well. Then, $dist_S( \mu _1(v), \mu _2(v') )$ is a distance between two ancestors of $\lambda (v)$ in *S*, which is maximized when one node maps to $\lambda (v)$ and the other maps to the root *r*(*S*) of *S* (noting that *v* and $v'$ can take either of these two roles).

We deduce that

$$\begin{aligned} d_{path}({\mathcal {G}}_1, {\mathcal {G}}_2) = \sum _{v \in V(G_1) \setminus L(G_1)} dist_S(\mu _1(v), \mu _2(m(v))) \le \sum _{v \in V(G_1) \setminus L(G_1)} dist_S( \lambda (v), r(S) ) \end{aligned}$$

where we note that we do not need to sum over the leaves of $G_1$, since corresponding leaves are mapped to the same species and do not contribute to the path distance.

We show that the value of $\sum _{v \in V(G_1) \setminus L(G_1)} dist_S( \lambda (v), r(S) )$ is always at most *H*(*S*). To do this, we build an injective function $\phi : V(G_1) \rightarrow V(S)$ such that for all *v* of $V(G_1)$, we have $\phi (v) \preceq _S \lambda (v)$. In other words, $\phi$ assigns each gene tree node *v* a *distinct* species that is equal or below its lca species.

We need a side argument first. For $x \in V(S)$, $\lambda ^{-1}(x)$ denotes the set of nodes *v* of $G_1$ such that $\lambda (v) = x$. Define $\#(x) = \sum _{y \preceq _S x} |\lambda ^{-1}(y)|$, the number of gene tree nodes whose lca-mapping is somewhere in *S*(*x*) (the subtree of *S* rooted at *x*). We claim that $\#(x) \le |V(S(x))|$. This is because if, for $v \in V(G_1)$, we have $\lambda (v) \preceq _S x$, then the clade *L*(*v*) of *v* only contains genes from species that descend from *x*. Thus, the subgraph of $G_1$ that contains only nodes that map to *x* or a descendant is a forest (a set of trees) with at most *L*(*x*) leaves (since each species appears only once). This is as many leaves as *S*(*x*), and because *S*(*x*) is binary, the aforementioned forest of $G_1$ cannot have more nodes than *S*(*x*), and thus $\#(x) \le |V(S(x))|$.

To construct $\phi$, consider each node of *V*(*S*) in a postorder fashion. We say that $x \in V(S)$ is “marked” if $\phi (v) = x$ for some $v \in V(G_1)$. Initially, all of *V*(*S*) is unmarked. We maintain the invariant that after handling some $x \in V(S)$, exactly $\#(x)$ vertices of *S*(*x*) are marked. If *x* is a leaf, take the unique $v \in L(G_1)$ with $\lambda (v) = x$ and put $\phi (v) = x$, which marks *x*. When arriving at *x* an internal node with children $x_1, x_2$, we assume that at most $\#(x_1) + \#(x_2)$ vertices of *S*(*x*) are marked before handling *x*. For each $v \in \lambda ^{-1}(x)$ in any order, put $\phi (v)$ equal to any unmarked node of *S*(*x*) so far. Note that is always possible, since the total number of nodes we want to mark is $\#(x_1) + \#(x_2) + |\delta ^{-1}(x)| = \#(x) \le |V(S(x))|$ and there are thus enough available nodes. Note, this maintains our invariant.

When this process ends at *r*(*S*), $\phi$ is injective and satisfies $\phi (v) \preceq _S \lambda (v)$ for all $v \in V(G)$. This means that each $\phi (v)$ is at equal or greater distance to *r*(*S*) than $\lambda (v)$, and so

$$\begin{aligned} \sum _{v \in V(G_1) \setminus L(G_1)} dist_S(\lambda (v), r(S)) \le \sum _{v \in V(G_1) \setminus L(G_1)} dist_S(\phi (v), r(S)). \end{aligned}$$

In the latter sum, we sum over internal nodes *v* of $G_1$, and so each $\phi (v)$ is a distinct *internal* node of *S* (they cannot be leaves because leaves of $G_1$ mark all leaves of *S*, so internal nodes of $G_1$ can only mark internal nodes of *S*). This latter sum is thus at most $\sum _{x \in V(S) \setminus L(S)} dist_S(x, r(S)) = H(S)$, because $\phi$ is injective.

Finally, we show that $H(S) \le (n-1)(n - 2)/2$ by induction on *n*. If $n = 1$ or $n = 2$, the longest path from *r*(*S*) to an internal node has length 0, which verifies the base case. So suppose $n \ge 3$. Let *x* be a deepest leaf of *S*, which must be part of a cherry, i.e., a leaf pair *x*, *y* that have a common parent *z*. Let $S - x$ denote the species tree in which leaf *x* is removed and its parent *z* of degree 2 suppressed (that is, remove *z* and add *y* as a child of its parent). By induction, $H(S - x) \le (n - 2)(n - 3)/2$. If we add back *x* and *z* to $S - x$ to recover *S*, the lengths of the previous root-to-internal node paths is unchanged, and we only add a path of length at most $n - 2$ from *r*(*S*) to *z* (in the worst case, that path goes through all the $n - 1$ internal nodes of *S*). We get $H(S) \le (n - 2)(n - 3)/2 + n - 2 = (n - 1)(n - 2)/2$. $\square$

We can now proceed to prove the following theorem.

### Theorem 3

Let *S* be a species tree on $n \ge 2$ leaves. Then

$$\begin{aligned} diam(d_{plr},S) = 2\alpha \cdot H(S) + (1 - \alpha )(2n - 2). \end{aligned}$$

Moreover, among all species trees with *n* leaves, the diameter is maximized when *S* is a caterpillar, in which case $diam(d_{plr}, S) = \alpha (n - 1)(n - 2) + (1 - \alpha )(2n - 2)$.

### Proof

We first show that our expression is an upper bound for the diameter. Let ${\mathcal {G}}_1 = (G_1, S, \mu _1, l_1)$ and ${\mathcal {G}}_2 = (G_2, S, \mu _2, l_2)$ be in ${\textsf{G}}^S$. For the label component of $d_{plr}$, since $G_1$ and $G_2$ have the same number of leaves, the maximum number of different nodes is bounded by the maximum number of internal nodes per tree. Given that every species has exactly one gene, this is exactly $n-1$. Hence, for the $d_{lbl}$ component, the cost is at most $2(n - 1) = 2n - 2$ considering both directions. As for the $d_{path}$ component, we know by Lemma 3 that the cost is at most *H*(*S*) in each of the two directions. This justifies the upper bound.

For an example that achieves this bound, suppose that $G_1$ and $G_2$ have the same topology as *S* (that is, they are both a copy of *S*, but we replace each leaf by a gene from that species). For $\mu _1$, we use the lca-mapping between $G_1$ and *S*, and put $l_1(v) = spec$ for every $v \in V(G_1) \setminus L(G_1)$ (which is possible since $G_1$ is a copy of *S* and uses the lca-mapping). For $G_2$, for every $v \in V(G_2) \setminus L(G_2)$, we put $\mu _2(v) = r(S)$ and $l_2(v) = dup$.

Note that because all internal node labels differ, this example achieves the maximum $d_{lbl}$ value possible. For the path component, let $s \in V(S) \setminus L(S)$. Let $v \in V(G_1)$ be the corresponding node in $G_1$ and $v'$ the corresponding node in $G_2$ (i.e., the copy of *s* in the trees). Note that $m(v) = v'$ and $m(v') = v$, and that $\mu _1(v) = s, \mu _2(v) = r(S)$. Hence, the contribution of *v* and $v'$ to the path component is $dist_S(s, r(S))$ on one side, plus $dist_S(r(S), s)$ on the other side. Since this holds for every internal *s* of *V*(*S*), the cost of the $d_{path}$ component is 2*H*(*S*).

As for the second part of the statement, first notice that by Lemma 3, *H*(*S*) is never more than $(n-1)(n-2)/2$, and so $diam(d_{plr}, S) \le \alpha (n-1)(n-2) + (1-\alpha )(2n - 2)$. Suppose that *S* is a caterpillar. Notice that the deepest internal node *X* satisfies $d(X, r(S)) = n - 2$ (there are $n - 1$ internal nodes in *S*, and the path goes through all of them). Then *p*(*X*) has a path of length $n - 3$ to *r*(*S*), then *p*(*p*(*X*)) of length $n - 4$, and so on, so that $H(S) = \sum _{i=1}^{n-2} i = (n - 1)(n - 2)/2$, achieving the maximum possible *H*(*S*). $\square$

### On the labeled RF distances

We now take a brief detour into another distance designed to compare reconciliations, namely the labeled Robinson-Foulds distances as presented in [55, 56], of which there are two variants. These distances are used in the next section and we briefly discuss upper bounds on their diameters. An edge of a tree is *internal* if none of its endpoints is a leaf. A *labeled tree* is a pair ${\mathcal {T}}= (T, l)$ where *T* is an unrooted tree without degree two nodes, and $l : V(T) \setminus L(T) \rightarrow X$ assigns some label from some set *X* to each internal node (one can think of the label set as $X = \{spec, dup\}$). A *label-flip* is an operation that changes the label of an internal node. An *extension* is the reverse of a contraction: it takes a node *v* and a non-empty subset *X* of its neighbors, creates a new node *w*, deletes the edges $\{vx : x \in X\}$, then adds the edges $\{wx : x \in X\}$ along with *vw*, such that the latter must be internal. A *labeled contraction* is an operation that contracts an internal edge *uv* satisfying $l(u) = l(v)$, and a *labeled extension* is an extension of *v* that creates node *w* with $l(w) = l(v)$.

Given two labeled trees ${\mathcal {T}}_1 = (T_1, l_1), {\mathcal {T}}_2 = (T_2, l_2)$, the *ELRF distance* [55] between ${\mathcal {T}}_1$ and ${\mathcal {T}}_2$ is the minimum number of labeled contractions, labeled extensions, and label-flips required to transform ${\mathcal {T}}_1$ into ${\mathcal {T}}_2$.

The *LRF distance* [56] is the minimum number of contractions, extensions, and label-flips required to transform ${\mathcal {T}}_1$ into ${\mathcal {T}}_2$ (note that the authors use the notion of node deletions and insertions, but are stated in [56] to be the same as contractions and extensions).

For an integer $n \ge 3$, the diameter of the ELFR (resp. LRF) distance is the largest possible distance among all possible labeled trees with *n* leaves. These diameters were not discussed in the literature. We provide bounds which we believe to be tight, under the assumption that the label set consists of two elements $X = \{spec, dup\}$.

**Fig. 4:**
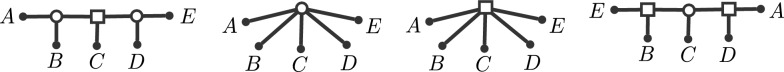
An example of two labeled trees (left and right), with $n = 5$ leaves and two internal edges, which both need to be contracted. To achieve this under the ELRF distance, we can perform $\lfloor (n - 2)/2 \rfloor = 1$ relabeling to make every label a circle (not shown), then contract every internal edge to obtain a star tree (second drawing). We can then change the remaining label, and reverse the operations to obtain the right tree. This takes $7 = 3n - 8$ operations.

#### Proposition 2

For $n \ge 3$ and label set *X* of size 2, the ELRF diameter is at most $3n - 8$. Furthermore, the LRF diameter is at most $2n - 5$.

#### Proof

Let ${\mathcal {T}}_1 = (T_1, l_1), {\mathcal {T}}_2 = (T_2, l_2)$ be two labeled trees with *n* leaves. Consider the ELRF distance first. Note that an unrooted tree has at most $n - 3$ internal edges (if we start with $n=3$ leaves, there are 0 such edges, and by adding one leaf at a time, we create at most one internal edge for each leaf added). We can make at most $n - 3$ contractions on $T_1$ to turn it into a star tree, i.e., a tree with a single internal node. However, contractions must have the same label on both endpoints, so we may need to make all the labels identical before doing so. There are at most $n - 2$ internal nodes in an unrooted tree.

If *n* is odd, we can always achieve the same label everywhere with $\lfloor (n-2)/2 \rfloor = (n - 3)/2$ label flips by changing the label that occurs the least frequently. Thus, with $(n - 3)/2 + n - 3$ operations, we can transform ${\mathcal {T}}_1$ into a star tree. We can do the same with ${\mathcal {T}}_2$, so one way of turning ${\mathcal {T}}_1$ into ${\mathcal {T}}_2$ is to make ${\mathcal {T}}_1$ a star tree, possibly flip the label of the internal node, then reverse the path from ${\mathcal {T}}_2$ to a star tree. Counting each step, this results in at most

$$\begin{aligned} (n-3)/2 + n - 3 + 1 + n - 3 + (n-3)/2 = 3n - 8 \end{aligned}$$

operations. If *n* is even, we have two cases. If there are $(n - 2)/2$ of each label in both ${\mathcal {T}}_1$ and ${\mathcal {T}}_2$, we see that $(n - 2)/2 + n - 3$ operations suffice to turn ${\mathcal {T}}_1$ into the star tree with either label, and the same holds for ${\mathcal {T}}_2$, and thus that $2((n-2)/2 + n - 3) = 3n - 8$ operations suffice. If, say, one label $x \in X$ occurs strictly more than $(n - 2)/2$ times in, say, ${\mathcal {T}}_2$, we can turn ${\mathcal {T}}_1$ into a star tree with $(n - 2)/2 + n - 3$ flips, flips the single label into *x* if needed, then perform $n - 3$ extensions, and then strictly less than $(n-2)/2$ relabels. This also results in at most $3n - 8$ operations.

The LRF bound uses a similar idea, except that contractions do not need to have their endpoint labels identical. We can thus perform at most $n - 3$ contractions on ${\mathcal {T}}_1$ to obtain a star tree, possibly flip the label of the internal node, and perform at most $n - 3$ extensions, adding the correct label each time, to obtain ${\mathcal {T}}_2$, resulting in $n - 3 + 1 + n - 3 = 2n - 5$ operations. $\square$

The intuition is that we can always contract all $n - 3$ internal edges of the first tree. In ELRF, we may have to relabel half of the $n - 2$ internal nodes to do this, so using $n - 3 + (n - 2)/2$ operations to reach a star tree (in the proof we show that this bound can be achieved while also attaining any desired label at the root of the star, with some case handling required for odd versus even *n*). This has to be reversed, leading to $3n - 8$. In LRF, we can just contract all $n - 3$ internal edges directly, possibly relabel the internal node of the star tree, then extend. It is possible that these bounds are tight. Consider Fig. 4 for the ELRF distance. If we generalize this pattern, it would appear that we need to flip $\lfloor (n-2)/2 \rfloor$ nodes, do $n - 3$ mandatory contractions, flip the central node, and reverse the process. This results in the upper bound $3n - 8$. For LRF, one can think of a pair of trees with no label in common, that require the maximum of $2n-6$ contractions and extensions, plus a label flip. However, proving that such examples cannot be handled better is not trivial, and since these distances are not the focus of the paper, we reserve those for future work.

## Methods and experiments

We compared the distribution of the PLR semi-metric against the classical Robinson-Foulds (RF) and its ELRF and LRF variants. To this end, we designed and implemented a stepwise procedure to simulate reconciled trees. The software tool to compute $d_{plr}$ is available as open source at: https://pypi.org/project/parle/.

### Simulation of reconciliations

#### Random scenarios on a fixed set of leaves

The existing programs for simulation of reconciliations like AsymmeTree or SaGePhy [66, 67] operate in a top-bottom fashion by first simulating ancestral genes/species followed by a birth-death process generating speciation, duplication, and loss events among others. This procedure does not guarantee trees with a fixed set of genes, whereas the PLR, LRF, and ELRF metrics require trees with the same set of leaves. To fulfill this requirement, we designed Algorithm 2, which takes as input a species tree *S*, as well as a set of genes $\Gamma$ and the assignment of species $\sigma :\Gamma \rightarrow L(S)$, then builds a reconciled gene tree over leafset $\Gamma$ in a bottom-up fashion. At each iteration it picks pairs of genes $x',x''\in \Gamma$ and substitutes them with a newly created node *x*, being the parent of the chosen genes. Finally, *x* is associated with an event and mapped to the species tree in Line 7. Algorithm 2 uses the lca-mapping $\mu$ for the generated gene trees. It is known that this map satisfies time-consistency, and that a node *x* with children $x', x''$ can be a speciation if and only if $\mu (x) \notin \{\mu (x'), \mu (x'')\}$ [3]. If this is not satisfied, the algorithm assigns $l(x) = dup$, and otherwise chooses $l(x) \in \{dup, spec\}$, which guarantees the *speciations separate species* condition.

Algorithm 2 considers a probability distribution *P* of picking $x',x''\in \Gamma$. In our implementation, this probability decays exponentially w.r.t. the distance between the species where $x'$ and $x''$ reside, in other words, the larger $d=dist_S(\mu (x'), \mu (x''))$ is, the smaller the chance of choosing $x',x''$. In particular, we use the probability $e^{-0.7 d}$. This approach is intended to prevent close elements in the gene tree from being mapped to distant nodes in the species tree, such a setting causes most of the inner nodes in the gene tree to be mapped near the root of the species tree, which would in turn create many *dup* nodes.

**Algorithm 2:**
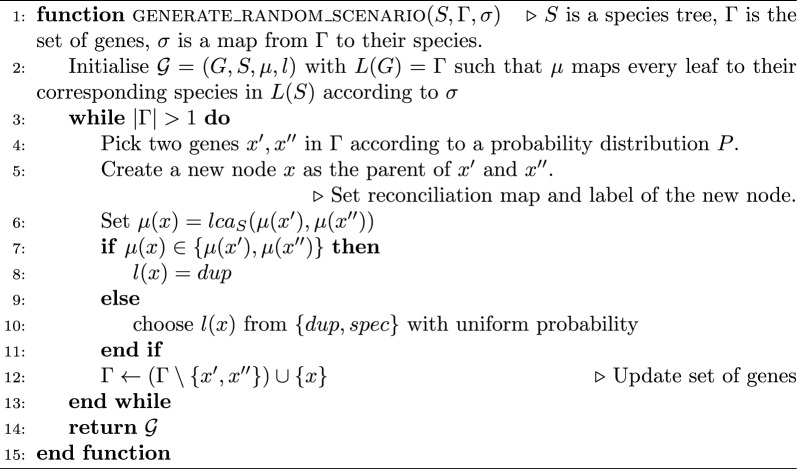
Simulation of random reconciliation scenarios

In total, we generated 9 sets of random reconciliations, obtained as follows. First, we generated three species trees $S_n$, where *n* is the number of leaves: $S_{10}$, $S_{25}$, and $S_{50}$, using the AsymmeTree package [66] under the *innovations model* as described in [68]. For each species tree $S_i$ we generated the gene sets $\Gamma _{i,5}$, $\Gamma _{i,10}$, and $\Gamma _{i,20}$, together with the assignments of species $\sigma _{i,5}$, $\sigma _{i,10}$, and $\sigma _{i,20}$ in such a way that for the set $\Gamma _{i,j}$ each species $y \in L(S_i)$ contains at least one gene and at most *j* genes. Considering this restriction, the number of genes for each species was chosen with uniform probability.

*Distance distribution and normalization* Given a set $R_{i,j}$ of random reconciliations generated from $S_i$ and $\Gamma _{i,j}$, we computed the PLR, ELRF, LRF, and RF measures for each pair of different reconciliations. We set $|R_{i,j}|=50$, resulting in 1225 total comparisons per set of random reconciliations. As argued in Section 2.2, the parameter $\alpha$ of PLR is aimed to balance the quadratic-versus-linear components of the distance. Following this analysis, we set $\alpha =1/i$ for the dataset $R_{i,j}$. Furthermore, to address the impact of $\alpha$ on the metric we also used the values 0, 0.25, 0.5, 0.75, and 1.

We normalized the distances obtained to have a fair comparison between the distribution of the different measures. We used two strategies, first, we normalized PLR by the theoretical diameter of the distance, while ELRF by its upper bond, and second by the empirical max normalization, which consists of dividing each computed value of a measure by the maximum encountered in the dataset for that measure.

### Analysis of dissimilarity distributions and diameters

#### Distributions with max-normalization

**Fig. 5:**
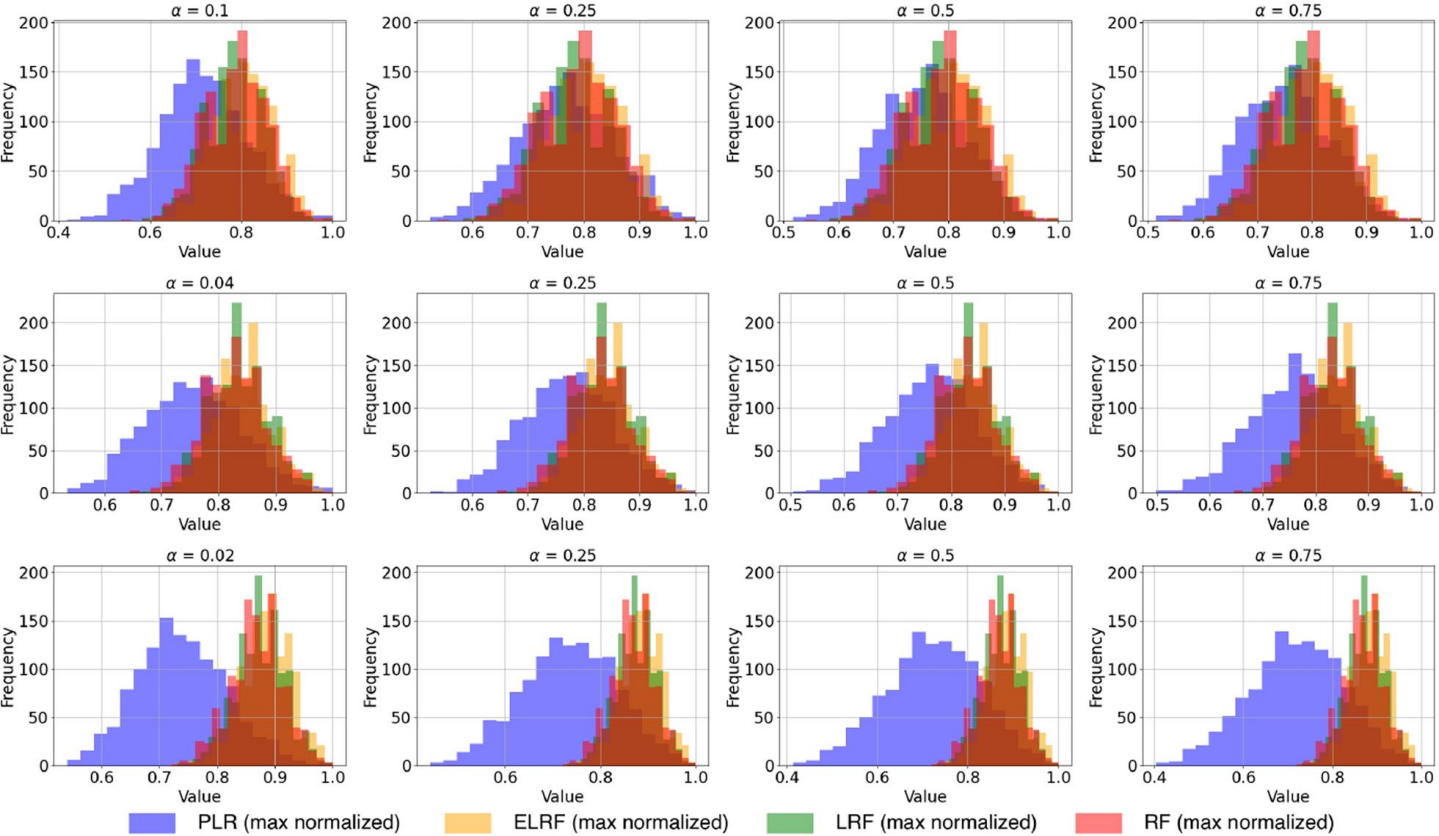
Distributions of the PLR, ELRF, LRF, and RF metrics for datasets $\Gamma _{10,20}$, $\Gamma _{25,10}$, and $\Gamma _{50,5}$, from top to bottom rows, respectively, and alpha values from the set $\{\frac{1}{n}, 0.25, 0.5, 0.75\}$, with *n* as number of species. Each row corresponds to a dataset, while each column represents a different value of $\alpha$. The *x*-axis represents max-normalized values ranging from 0 to 1, and the *y*-axis is the frequency of these values. The PLR measure in purple shows a centered and symmetric distribution with a broader spread. The ELRF, LRF, and RF metrics, shown in light orange, green, and red, respectively, exhibit right-skewed distributions towards the higher end of the scale.

Each subplot of Fig. 5 shows four distributions comparing the PLR, ELRF, LRF, and RF metrics represented in blue, light orange, green, and red, respectively.

The ELRF, LRF, and RF distributions exhibit right-skewness, indicating that many data points cluster towards higher values. This skewness suggests a higher frequency of larger distances, a common trait among these metrics. Notably, the RF metric often shows smaller distances because it ignores label changes, whereas the ELRF and LRF metrics yield almost identical values, performing very similarly, as expected.

In contrast, the PLR distribution is centered around its mean, displaying a broader spread of measurements. This symmetric distribution indicates that the PLR metric has a greater variability in distance measurements, highlighting its sensitivity, that is, a balanced penalization of all the elements of an evolutionary scenario. This contrasts with the more concentrated and nearly identical distributions of ELRF, LRF, and RF.

#### The theoretical diameter is hard to reach

**Fig. 6:**
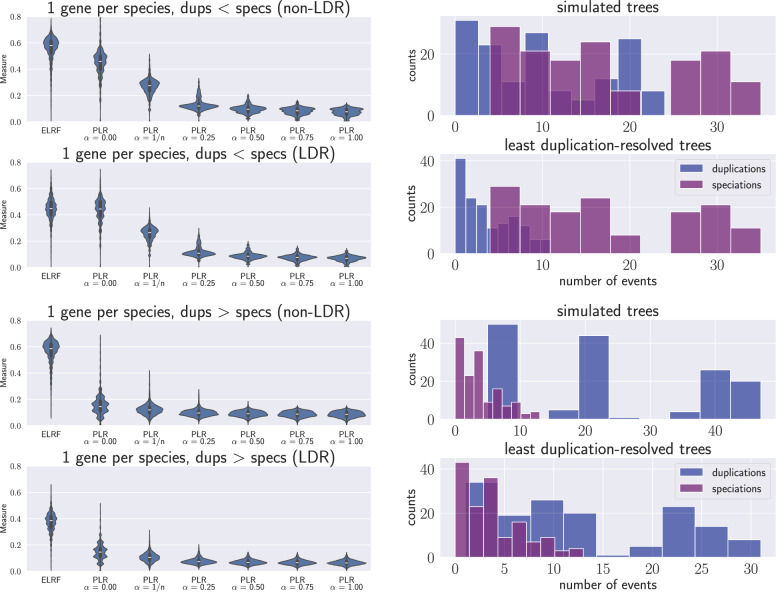
Comparison of the distribution of ELRF and PLR measures with different values for the parameter $\alpha$, and different proportions of duplication/speciation events. The measures are shown for both the least duplication-resolved trees (LDR) and non-LDR. All the plots consider reconciliations with 10, 25, and 50 species. The parameter $\alpha =1/n$ aims to balance the linear-vs-quadratic components of the distance, where *n* is the number of species. Note that the biggest change in the distribution of the PLR measure happens for small values of $\alpha$.

Figure 6 presents the distribution of the ELRF distance and the PLR distance for various values of the parameter $\alpha$. We omit the plots for LRF and RF distances since they closely resemble the ELRF distributions, as discussed in the previous section.

The first two rows in Fig. 6 compare trees with fewer duplications than speciations, while the subsequent rows involve trees with an equal or greater number of duplications compared to speciations. The PLR measure is normalized by the theoretical diameter introduced here, whereas the ELRF is normalized by its upper bound. Note that ELRF consistently has higher values than PLR and that these values are significantly far from the theoretical diameter. The shape of the PLR distribution remains largely unchanged as $\alpha$ increases, likely due to the diminishing contribution of the linear component relative to the quadratic component as $\alpha$ grows. On the right side of the figure, we observe the frequency of speciation and duplication events in our simulated reconciled trees, as well as their least duplication-resolved (LDR) counterparts. Notably, when there are more speciations than duplications, the PLR measure increases but still remains far from the theoretical diameter.

**Fig. 7:**
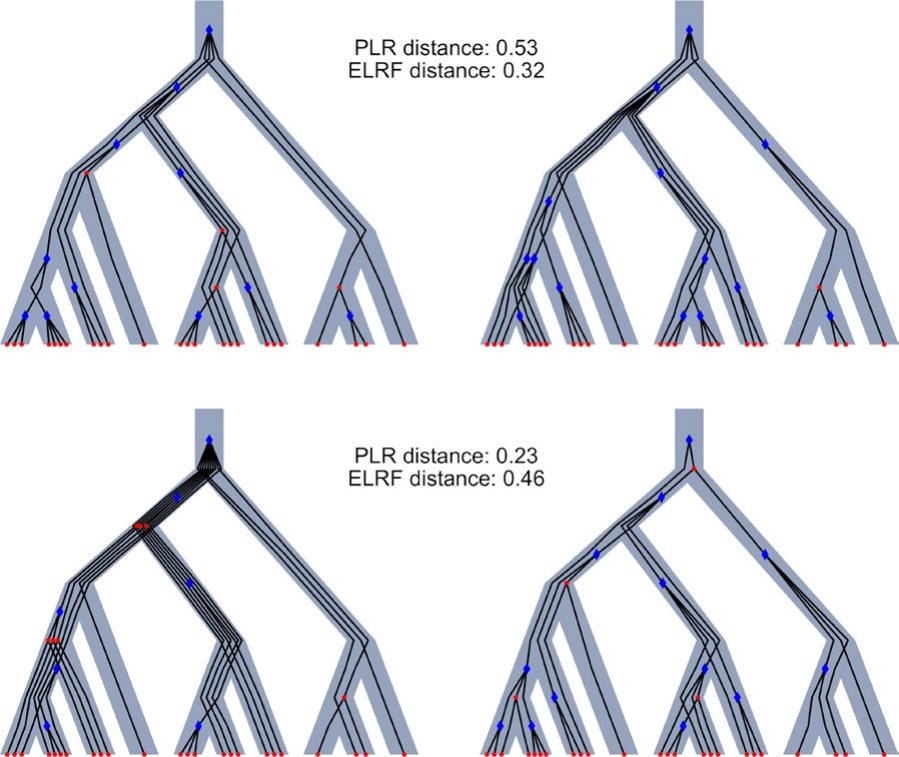
Examples of distance between reconciliations and gene trees, plotted using REvolutionH-tl [69]. The reconciliations have 10 species and 24 genes, with $\alpha =1/10$. The upper row has a large PLR value but a small ELRF distance. In contrast, the bottom row shows trees when PLR is small even when ELRF is big.

Figure 7 illustrates important differences between the measures, since we can observe two different scenarios: (1) where ELRF is significantly smaller than PLR, suggesting that reconciliations may be completely different even when gene tree topologies are similar; and (2) conversely, PLR may be significantly small when the ELRF is large, suggesting that different gene tree topologies could have similar reconciliations.

### Evaluation of the best tree rootings

One of the purposes of a measure for reconciled gene trees is to evaluate, among a set of competing gene trees inferred by different approaches, which prediction is the closest to a gold standard tree. In this context, we study the problem of *rooting* an unrooted gene tree, and view the set of all possible rootings as the set of competing hypotheses. We then compute the PLR and LRF distances against a true simulated gene tree and check whether the two measures agree or not on which rooting is the “best”, according to the distance. We emphasize that identifying the correct root can be challenging, and several methods such as outgroup selection and midpoint rooting have been widely studied. Misplaced roots can lead to incorrect evolutionary interpretations, affecting downstream analyses such as reconciliation and event inference.

To simulate evolutionary scenarios, we again utilized the Asymmetree software, which can also generate synthetic gene sequences under user-specified evolutionary models. The following parameters were configured:

- For each $n \in \{5,10,15,20,25,30,35,40,45,50\}$, we generated 100 species trees with *n* leaves, choosing for each an evolutionary model among the available ones. The available models comprise a pure-birth Yule [70], constant-rate birth-death process [71], episodic birth-death process [72] and innovations [73].
- For every species tree *S*, we generated 1 gene tree with duplication rate 1.0, loss rate 1.0 and horizontal gene transfer rate 0.0 and we generated amino acid sequences of length 500 using the substitution model JTT [74].

We will refer to the set of gene trees and simulated sequences and their corresponding species trees as a synthetic reconciliation *dataset*, and we will denote by $D_n$ the set of pairs $(S, {\mathcal {G}})$ generated, such that *S* is a species tree with *n* leaves and ${\mathcal {G}}$ the corresponding simulated gene tree (along with its reconciliation and sequences). The simulated gene sequences were used to reconstruct gene trees using RAxML [75], employing the amino acid model with default parameters. The gene trees inferred from these sequences were unrooted, and our goal was to evaluate the “best” rooting according to our measures.

For every inferred gene tree, we then performed the following analysis: for every edge, we subdivide the edge to create a new node, and we assign the new node as the root of the tree. We will refer to the resulting tree as a *rooting*. We generated every possible rooting of the tree, so that an unrooted gene tree with *m* edges would result in *m* rooted trees to analyze. Each rooting was reconciled with its corresponding known species tree using the traditional lca-mapping as described in [76]. These functions are available at https://github.com/AliLopSan/lcamap.git.

Then, for each rooting we computed the PLR and LRF distances with the true gene tree (we did not consider ELRF as its implementation can only approximate distances). The rooting that minimizes the distance to the simulated tree is seen as the best rooting. More often than not, we observed ties in best rootings, so we computed the number of best candidates for each distance. Therefore, for a true simulated reconciled gene tree ${\mathcal {G}}$ and an inferred unrooted gene tree *T*, we define:

- $Best(T, d_{plr})$ as the set of reconciled rootings $T'$ of *T* that minimize $d_{plr}(T', {\mathcal {G}})$;
- $Best(T, d_{lrf})$ as the set of reconciled rootings $T'$ of *T* that minimize $d_{lrf}(T', {\mathcal {G}})$, which is the LRF distance between $T'$ and *G*.

**Fig. 8:**
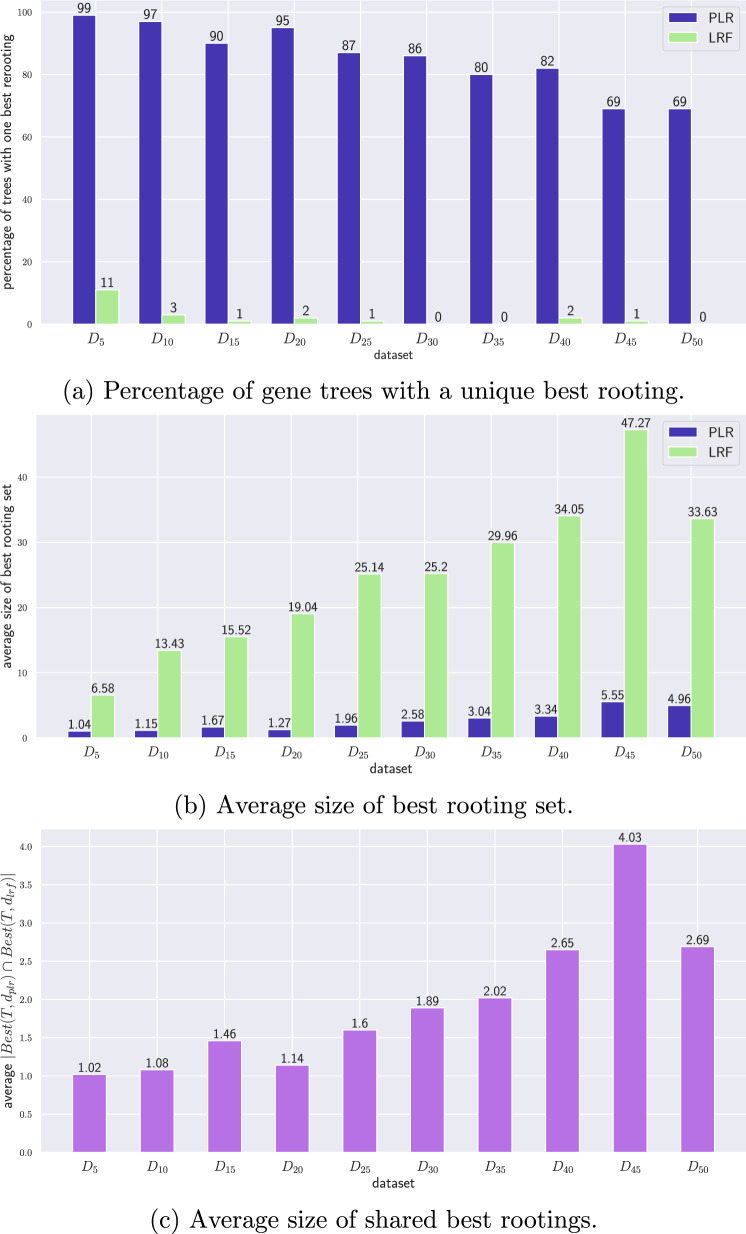
Analysis of best rootings sets. In (**a**) we compute the percentage of trees such that $|Best(T, d_{plr})| = 1$, and such that $|Best(T, d_{lrf})|=1$. In (**b**) we are interested in the average size of this set, and (**c**) shows the average size of the intersection $Best(T, d_{plr}) \cap Best(T,d_{lrf})$.

These results are summarized in Fig. 8. In subfigure (a), we observe a significant difference in the ability of $d_{plr}$ to discriminate a single best rooting, as the majority of gene trees in every synthetic reconciliation dataset possess one. The percentage of trees with a unique best tree remain relatively high even as synthetic reconciliation dataset size increases (e.g., from 99% for $S_5$ to 69% for $S_{50}$). This contrasts with LRF, which identifies a unique best rooting in only a small fraction of cases. One possible explanation is that different rootings generate different duplication and speciation events, as well as different gene-species maps. Since the LRF distance can only vary when the duplication/speciation events differ, it does not account for topological differences between rootings or changes in the species map, leading to ties and a larger best rooting set. In contrast, by incorporating all this information, $d_{plr}$ achieves greater discriminatory power, better evaluating which rooting is closest to the true tree. We do note that for $d_{plr}$ the percentage of trees with a unique best rooting generally decreases with *n*, the number of leaves in the species tree. This is expected, as larger species trees usually yield larger gene trees, which in turn have a greater number of possible rootings and more opportunities for ties.

Subfigure (b) shows the average size of the best rooting set, i.e., $\frac{1}{|{\mathcal {T}}|} \sum _{T \in {\mathcal {T}}} |Best(T, d)|$, where ${\mathcal {T}}$ is the set of inferred gene trees in the synthetic reconciliation dataset and $d \in \{d_{plr}, d_{lrf}\}$. The results indicate that PLR maintains a much smaller average size for the best rooting set across all synthetic reconciliation datasets. For $S_5$, the average size is close to 1.0, indicating that most trees have a single best rooting. As the synthetic reconciliation dataset size increases, this average gradually rises, reaching a maximum of 5.55 for $S_{45}$. In contrast, LRF exhibits a much larger average size for the best rooting set, increasing substantially as dataset size grows (e.g., 6.58 for $S_5$ to 47.27 for $S_{45}$). The comparison shows that even when ties are present under the $d_{plr}$ measure, the quantity of retained candidates is small.

Finally, subfigure (c) shows the average size of the intersection of best rootings found by $d_{plr}$ and $d_{lrf}$. In all synthetic reconciliation datasets, the average size of this intersection is smaller than the average size of $|Best(T, d_{plr})|$. This indicates that there are several cases where the symmetric difference between the best sets is non-empty, i.e., $d_{plr}$ ranks some rooting first that $d_{lrf}$ does not, and vice versa. Therefore, different choices of measures may favor different reconstruction methods. In future work, we plan to investigate when and how such differences may arise.

### Important differences between PLR and LRF

The Path-Label Reconciliation (PLR) measure and the Labeled Robinson-Foulds (LRF) metric both compare gene trees based on topology and evolutionary event labels but differ fundamentally in the aspects of tree structure they capture.

**Fig. 9:**
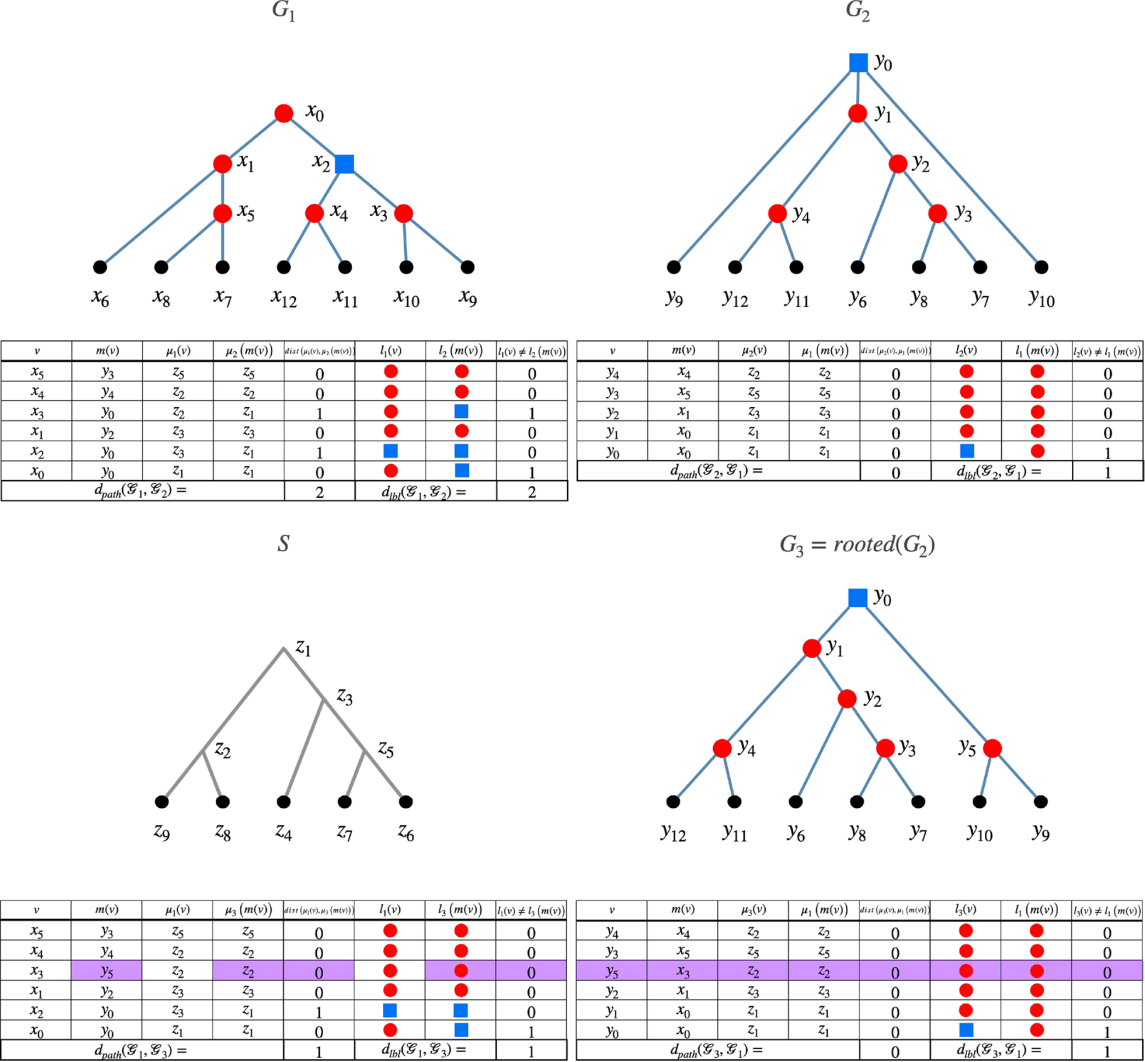
The tree $G_1$ represents the observable gene tree, while $G_2$ is an inferred gene tree with a polytomy at node $y_0$. Tree $G_3$ is the midpoint-rooted version of $G_2$, and *S* is the species tree. Event labels are shown as red circles (speciations) and blue squares (duplications). Black dots denote extant genes in $G_1$, $G_2$, and $G_3$ and extant species in *S*. The tables, arranged top to bottom and left to right, depict $d_{asym}({\mathcal {G}}_1, {\mathcal {G}}_2)$, $d_{asym}({\mathcal {G}}_2, {\mathcal {G}}_1)$, $d_{asym}({\mathcal {G}}_1, {\mathcal {G}}_3)$, and $d_{asym}({\mathcal {G}}_3, {\mathcal {G}}_1)$. Purple entries highlight information unique to $G_3$ due to its rooting, illustrating how PLR distance tends to minimize in $G_3$. Note that $\alpha$ is excluded from these calculations.

First, in terms of gene-species mapping, PLR considers gene-species mapping discrepancies that LRF ignores, accounting for the distances between species nodes to which corresponding gene nodes are mapped. When gene-species mappings between trees diverge significantly, these differences increase the PLR measure. In contrast, LRF focuses solely on topological differences and the labels of internal nodes, disregarding gene-species mappings. For example, if multiple gene tree nodes in different species of $G_1$ all map to the same node of $G_2$ under $m_{12}$, the path component can contribute significantly to $d_{plr}$, which is not considered by $d_{lrf}$. We can see an example that accounts for these differences in Fig. 9.

Second, when considering polytomies PLR may assign a greater distance than LRF, because non-binary trees can often be transformed with fewer operations of contractions, extensions, or label adjustments. In contrast, PLR evaluates distances between mapped gene nodes in the species tree and captures differences in evolutionary events between corresponding gene nodes, regardless of tree topology.

Finally, in smaller trees LRF often requires fewer operations to transform one tree into another due to the limited number of internal nodes, which simplifies topological comparisons. PLR, however, still accounts for gene-species mapping distances, which may not reduce as efficiently in smaller trees, potentially resulting in a higher PLR distance.

## Conclusion

In this work, we have underscored the unique attributes of PLR, a novel semi-metric designed for comparing reconciled gene trees within a fixed species tree framework. Unlike existing metrics such as RF, LRF, and ELRF, which primarily focus on tree topology, PLR incorporates all elements of an evolutionary scenario: a species tree, gene trees, speciation/duplication labeling and a mapping from gene trees to species tree. This broader scope provides a more holistic measure of dissimilarity between reconciled gene trees, offering researchers a nuanced understanding of evolutionary relationships.

One notable advantage of PLR is its flexibility, particularly regarding the parameter $\alpha$, which allows users to balance the quadratic and linear components of the distance according to their specific research context. This flexibility enhances the metric’s applicability across diverse evolutionary scenarios, providing researchers with a customizable tool for reconciliation analysis. Additionally, our experiments reveal that PLR exhibits a symmetric and broadly spread distribution around its mean, indicating sensitivity to variations in reconciliations and finer granularity in distinguishing between different tree pairs. Despite its strengths, PLR does have some limitations. For instance, while the flexibility of $\alpha$ is advantageous, it also introduces a degree of subjectivity into the metric’s application, as users must determine the appropriate value for their specific context. Moreover, our theoretical analysis highlights a large theoretical diameter for PLR, which is seldom reached in practice. Tighter bounds are needed to improve practical applicability and interpretability. One of the key strengths of PLR is its computational efficiency, with an *O*(*n*) time complexity. This efficiency is particularly beneficial for analyzing large datasets or trees, where computational resources and time are critical constraints.

Looking ahead, future directions for PLR include refining the theoretical bounds of its diameter. An important theoretical problem that remains open is determining whether *binary* gene trees satisfy the triangle inequality. Additionally, developing metrics between gene trees with different leaf sets would significantly broaden its applicability. Incorporating alternative methods for matching ancestral genes, such as those proposed by Lin et al. [57], or using asymmetric cluster affinity as suggested by Wagle [53], could further enhance the metric’s accuracy and relevance.

In conclusion, PLR represents a significant advancement in the comparison of reconciled gene trees, offering a detailed and flexible measure of dissimilarity. Its computational efficiency and comprehensive event consideration make it a valuable tool for evolutionary studies, with potential for further refinement and application in future research.

## Data Availability

The computational results are available at: https://pypi.org/project/parle/ and also at https://github.com/AliLopSan/lcamap . The full datasets used in this study can be found at: https://gitlab.com/jarr.tecn/recondist.
